# Postoperative outcomes of preoperative exercise training in patients with operable non-small cell lung cancer: a systematic review and meta-analysis

**DOI:** 10.3389/fonc.2025.1563478

**Published:** 2025-09-12

**Authors:** Cuifang Li, Haidan Meng, Ye Wei, Yugan Liang, Yangqian Xu, Xiaomeng Huang, Weiming Liang, Jieru Quan, Shanguang Wu, Xueyan Wei

**Affiliations:** ^1^ The First Affiliated Hospital of Guangxi University of Science and Technology, Guangxi University of Science and Technology, Liuzhou, Guangxi, China; ^2^ School of Economics and Management, Guangxi University of Science and Technology, Liuzhou, Guangxi, China; ^3^ Guangxi College Key Laboratory of Innovation Research on Medical and Engineering Integration, Guangxi University of Science and Technology, Liuzhou, Guangxi, China

**Keywords:** lung cancer, lung resection, exercise training, complication, pulmonary function, exercise capacity, meta-analysis

## Abstract

**Introduction:**

This meta-analysis was designed to compare the outcomes of preoperative exercise training versus no preoperative exercise for lung cancer patients scheduled for lung resection.

**Materials and methods:**

Four databases (Medline, Embase, Web of Science, and CENTRAL) were searched for randomized controlled trials (RCTs) comparing preoperative exercise training versus no preoperative exercise for lung cancer patients scheduled for lung resection. The primary outcomes were postoperative complications and postoperative length of hospital stay. The secondary outcomes included post-intervention pulmonary function, severe postoperative complications, postoperative 30-day mortality, postoperative duration of chest tube drainage, post-intervention dyspnea, and post-intervention health-related quality of life (HRQoL).

**Results:**

A total of 16 RCTs with 1,022 individuals were included in this meta-analysis. Compared with no preoperative exercise, preoperative exercise training significantly reduced the postoperative complications (OR = 0.33, 95%CI: 0.24 to 0.46, P < 0.0001) and postoperative length of hospital stay (95%CI: −3.11 to −1.40, P < 0.0001). In addition, preoperative exercise training significantly improved forced expiratory volume in 1 s (FEV_1_%) of predicted norm values (95%CI: 5.30 to 8.10, P < 0.0001), forced vital capacity (FVC%) of predicted norm values (95%CI: 1.90 to 4.23, P < 0.0001), peak expiratory flow (PEF) (95%CI: 12.44 to 60.93, P = 0.003), and peak oxygen uptake (VO_2peak_) (95%CI: 2.41 to 4.17, P < 0.0001), while reducing severe postoperative complications (OR = 0.35, 95%CI: 0.21 to 0.56, P < 0.0001) and post-intervention dyspnea (95%CI: −0.61 to 0.04, P = 0.02). There was no significant difference between the two groups regarding FEV_1_, FVC, carbon monoxide diffusing capacity (DLCO), six-minute walk distance (6MWD), postoperative 30-day mortality, postoperative chest tube drainage time, and post-intervention HRQoL.

**Conclusions:**

This meta-analysis indicated that preoperative exercise training was effective for lung cancer patients scheduled for lung resection, potentially reducing postoperative complications and hospital stay duration, while improving post-intervention pulmonary function and exercise capacity.

**Systematic Review Registration:**

https://www.crd.york.ac.uk/prospero/, identifier CRD42024607156.

## 1 Introduction

Lung cancer is the leading cause of cancer-related morbidity and mortality, responsible for around 2.5 million new cases and over 1.8 million fatalities globally. It accounts for approximately one in eight (12.4%) cancer diagnoses worldwide and one in five (18.7%) cancer-related deaths. The disease ranks first in incidence and mortality among men and second among women (1, 2). Surgery often leads to postoperative complications, which prolong hospitalization, increase the probability of admission to the critical care unit, and elevate mortality rates during the perioperative period (3). Postoperative outcomes are affected by multiple factors, including the type of surgical procedure, cancer stage, gender, and neoadjuvant medications; however, emerging evidence suggests that patients’ physical functions are pivotal. Pulmonary function and cardiorespiratory fitness prior to surgery have been recognized as predicting factors for postoperative complications and overall survival in lung cancer patients (4).

Exercise training is a systematic, organized, and repetitive kind of physical activity designed to enhance or sustain physical fitness as a primary or secondary objective (5). Studies demonstrated that exercise training enhanced functional and cardiorespiratory fitness (CRF) in persons with chronic obstructive pulmonary disease (6–8). This comprehension has led to the integration of exercise training into the preoperative therapy of patients scheduled for lung resection owing to lung cancer, with the objective of improving physical fitness to overcome the physiological stress caused by surgery, hence reducing postoperative morbidity and mortality. Multiple worldwide guidelines have been established to mandate specific perioperative cardiopulmonary exercise testing prior to the initiation of preoperative exercise training (9), in order to improve patients’ physical function and better manage the homeostatic disruption and stress response associated with surgery (10). Preoperative exercise training in patients slated for lung resection aims to improve health, namely, aerobic fitness, during the period between diagnosis and surgery, thereby reducing the risk of complications and decreasing hospital length of stay (LoS) (11). Preoperative exercise training has shown a decrease in hospitalizations and postoperative complications in patients following lobectomies or lung resections (12).

A previous meta-analysis indicated that higher preoperative cardiorespiratory fitness was associated with a reduction in postoperative pulmonary complications (13). Preoperative sarcopenia, characterized by diminished skeletal muscle mass and strength, might negatively impact postoperative outcomes, including complications and overall survival in colorectal, esophageal, pancreatic, and bladder cancers (14). Sarcopenia can develop into frailty, characterized by diminished reserve and resistance to stressors due to cumulative losses in numerous organ systems, resulting in an increased prevalence of unfavorable consequences, which is an independent risk factor for surgical complications, extended hospital stay, and fatality (15–18). Therefore, it is clear that enhancing the functional and physiological capacities of individuals is crucial for their ability to withstand stressful events like surgery and to promote recovery afterward (19). Postoperative complications are common in elderly individuals with low physical fitness, physical inactivity, malnutrition, and tobacco-related comorbidities (2, 20–22).

The available data for preoperative exercise training for patients with lung cancer are somewhat restricted. The previous systematic review on this subject demonstrated that preoperative exercise training decreased the incidence of postoperative complications, decreased LoS, and enhanced postoperative exercise capacity (23). Yet, this conclusion was derived from a mere 10 studies. More recently, another meta-analysis has yielded comparable findings (24). Regrettably, the conclusions were constrained by methodological limitations: four of the 16 studies included were not RCTs, which might have resulted in bias. Therefore, we conducted an updated meta-analysis that exclusively included RCTs, with the aim of providing clearer insights into the outcomes of patients with lung cancer who received preoperative exercise training and informing clinical decision-making.

## 2 Materials and methods

### 2.1 Search strategy

The present meta-analysis carefully followed the guidelines established by the Preferred Reporting Project for Systematic Review and Meta-Analysis (PRISMA) 2020 guidelines. The study has been formally registered at PROSPERO with the designation number CRD42024607156. A systematic search was conducted in four databases, namely, PubMed, Embase, Web of Science, and the Cochrane Library, to identify literature items published up to July 22, 2024. The search strategy used a combination of MeSH and free-text words following the PICOS principle. The search keywords were “lung cancer” AND “preoperative exercise” AND “randomized controlled trial”. **Supplementary Tables** provided a comprehensive listing of the search results.

### 2.2 Inclusion and exclusion criteria

The inclusion criteria were as follows: (1) Patients diagnosed with lung cancer who were about to undergo lung resection. (2) Patients in the intervention group received preoperative exercise training. The exercise sessions may be supervised, unsupervised, or a combination of both and can encompass aerobic, resistance, high-intensity interval or respiratory muscle training, or a combination thereof. (3) Patients in the control group received no preoperative exercise. (4) At least one of the following outcomes were reported: postoperative complications, postoperative length of hospital stay, post-intervention pulmonary function by FEV_1_, FVC and FEV_1_% of predicted norm values, FVC% of predicted norm values, PEF, DLCO, post-intervention exercise capacity measured by 6MWD and VO2peak, severe postoperative complications, postoperative 30-day mortality, postoperative chest tube drainage time, post-intervention dyspnea, and post-intervention HRQoL. (5) Study design: RCTs.

The exclusion criteria were as follows: (1) Other types of articles, such as case reports, publications, letters, reviews, editorials, pharmacological intervention, animal trials, and protocols. (2) Not relevant. (3) Full text not available. (4) Duplicate patient cohort. (5) Failed to obtain data.

### 2.3 Selection of studies

Selection of studies, including elimination of duplicates, was undertaken using EndNote (Version 20; Clarivate Analytics). An initial search was undertaken by two reviewers who independently deleted duplicate entries, assessed the titles and abstracts for relevance, and classified each study as either included or excluded. The settlement was arrived at through the attainment of consensus. A third author of the review would take on the role of an arbitrator if lacking a consensus.

### 2.4 Data extraction

Two separate reviewers conducted a thorough examination of the title and abstract, subsequently engaging in an exhaustive review of the entire text. A third reviewer was consulted to resolve the inconsistencies. Publication year, country, first author, sample size (preoperative exercise training group and no preoperative exercise group), study design, age, sex, current smoker, Non-Small Cell Lung Cancer (NSCLC) stage, American Society of Anesthesiologists (ASA) status, postoperative complications, postoperative length of hospital stay, post-intervention pulmonary function by FEV_1_ (25), FVC and FEV_1_% of predicted norm values (26, 27), FVC% of predicted norm values, PEF (28), DLCO (29), post-intervention exercise capacity measured by 6MWD (30) and VO_2peak_ (31, 32), severe postoperative complications, postoperative 30-day mortality, postoperative chest tube drainage time, post-intervention dyspnea, and post-intervention HRQoL were all extracted. The postoperative complications assessed with the Clavien-Dindo classification (33) score ≥2 were classified as severe postoperative complications. HRQoL was evaluated using EORTC-QLQ-C30, a disease-specific health-related quality of life (QOL) scale ranging from 0 to 100, wherein a higher score reflects either better function or worse symptomatic effect (34, 35).

### 2.5 Risk of bias assessment

Two independent reviewers assessed the risk of bias using the Cochrane Risk of Bias tool, which has seven domains: random sequence generation, allocation concealment, blinding of participants and personnel, blinding of outcome assessment, incomplete outcome data, selective reporting, and other bias. Group discussions were employed to resolve disputed results and correct discrepancies.

### 2.6 Data analysis and statistical methods

EndNote (Version 20; Clarivate Analytics) was used for article selection and duplication removal. The Cochrane Collaboration in Oxford, UK’s Review Manager 5.3 was used to analyze all study results. With a 95% confidence interval (CI), odds ratios (OR) were used to compare binary variables. A 95% CI was used to compare continuous variables. The medians and interquartile ranges of the continuous data were converted into corresponding means and standard deviations. The Cochrane Q p value and I^2^ statistic were used to evaluate the heterogeneity of each meta-analysis. A fixed-effect model (FEM) was used for low heterogeneity (I^2^ < 50%), and a random-effect model (REM) was used for high heterogeneity (I^2^ ≥ 50%) when analyzing pooled data. Using a traditional chi-square test, the statistical heterogeneity was assessed and shown to be statistically significant at a significance level of P < 0.05. The funnel plots’ visual evaluation was used to determine whether publication bias was present.

## 3 Results

### 3.1 Literature search

**Figure 1:**
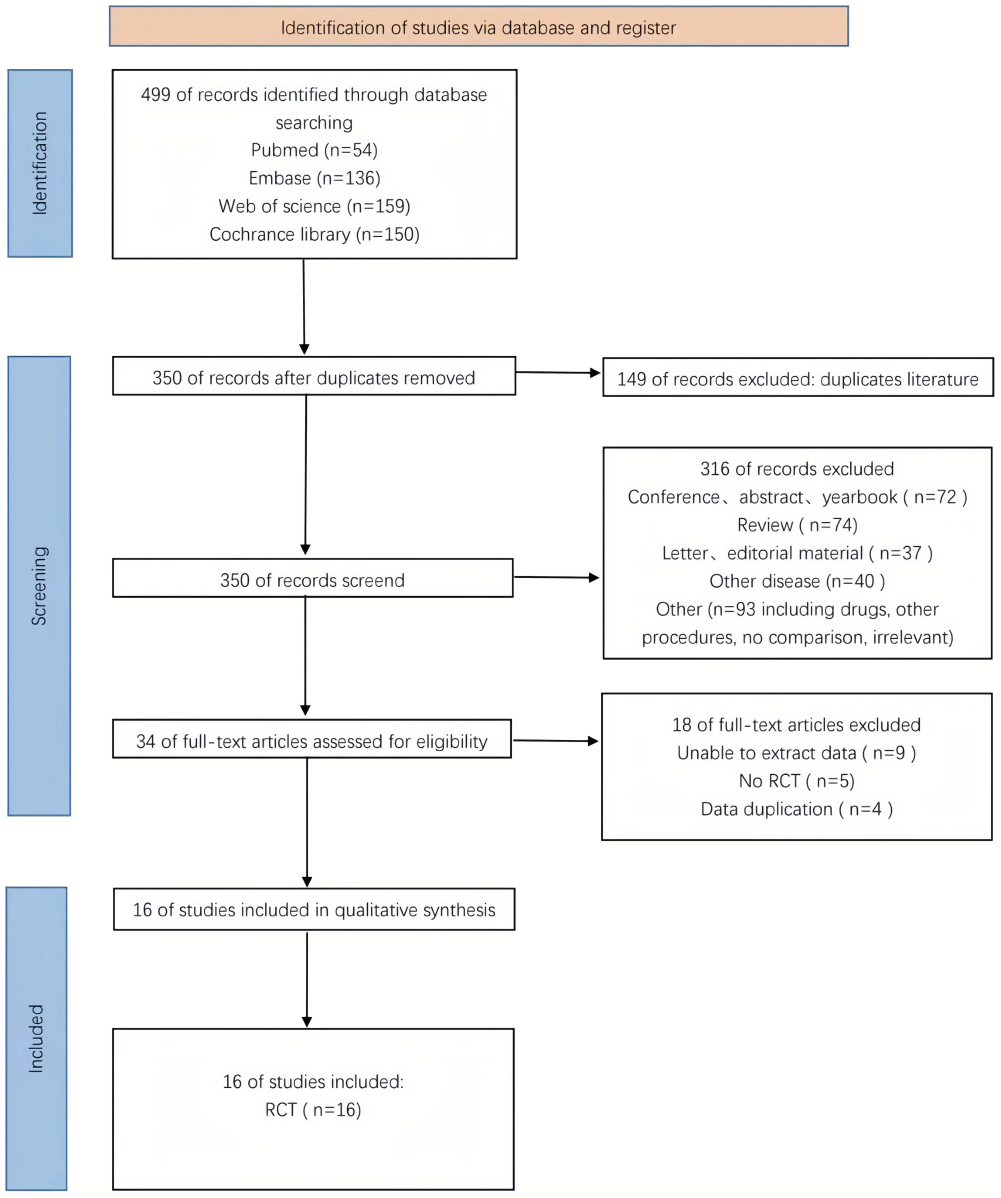
Flowchart of literature search strategies.

**Figure 1** illustrates the procedure of selecting and integrating literature. The initial search approach facilitated the identification of 499 potential research studies. A total of 23 papers fulfilled the criteria and were evaluated for potential inclusion following the examination of titles and abstracts. Finally, 16 RCTs were included in this meta-analysis following a comprehensive review of the full text (12, 28, 29, 36–48).

### 3.2 Characteristics of the included studies and quality assessment

The meta-analysis comprised 16 trials including 1,022 individuals, with 524 allocated to the preoperative exercise training group and 498 to the no preoperative exercise group. The registration ID, country, number, age, mean age, smoker, FEV1% of predicted norm values, NSCLC stage, ASA status, gender, and intervention are presented in **Table 1**.

**Table 1:** Patient characteristics of included studies and patients.

Author, year	Registration id	Country	Group	No.	Mean age, year	Current smoker, n	FEV1%	NSCLC stage		ASA status		Male%	Intervention
								I-II, n	III-IV, n	I-II, n	III, n		
Benzo 2011 (36)	NA	United States	E	10	70.20	1	43.40	NA	NA	NA	NA	50.00	1 week, inspiratory muscle training + endurance training
			U	9	72.00	2	52.10	NA	NA	NA	NA	44.00	No preoperative exercise
Pehlivan 2011 (37)	NA	Turkey	E	30	54.10	NA	65.40	NA	NA	NA	NA	NA	1 week, chest physiotherapy, and aerobic exercises + routine physical therapy
			U	30	54.76	NA	77.04	NA	NA	NA	NA	NA	No preoperative exercise
Stefanelli 2013 (29)	NA	Italy	E	20	65.50	NA	57.40	20	0	NA	NA	NA	3 weeks, high-intensity training + respiratory exercises
			U	20	64.80	NA	57.60	20	0	NA	NA	NA	No preoperative exercise
Fang 2013 (38)	NA	China	E	22	64.10	20	45.10	17	5	NA	NA	95.50	2 weeks, high-intensity training + respiratory exercises
			U	22	64.80	19	43.40	19	3	NA	NA	95.50	No preoperative exercise
Tereza 2014 (39)	RBR-3nm5bv	Brazil	E	12	65.00	10	48.00	11	1	NA	NA	33.33	4 weeks, strength and endurance training + inspiratory muscle training
			U	12	69.00	9	49.00	9	3	NA	NA	41.67	no preoperative exercise
Lai 2016 (40)	ChiCTR1900059756	China	E	24	63.13	7	NA	21	3	NA	NA	62.50	1 week, endurance training + inspiratory muscle training
			U	24	64.04	7	NA	21	3	NA	NA	54.20	No preoperative exercise
Huang 2017 (41)	ChiCTRIOR-16008109	China	E, arm a	30	63.00	7	NA	26	4	27	3	66.70	1 week, aerobic endurance exercise + inspiratory muscle training
			E, arm b	30	64.10	6	NA	24	6	27	3	70.00	1week, inspiratory muscle training
			U	30	63.60	7	NA	28	2	28	2	70.00	no preoperative exercise
Che 2017 (42)	ChiCTR1600045568	China	E	30	72.50	6	NA	26	4	28	2	53.30	1 week, aerobic endurance training + inspiratory muscle training
			U	30	71.60	5	NA	28	2	27	3	60.00	No preoperative exercise
Su 2017 (43)	ChiCTR1700022451	China	E	51	63.80	32	NA	44	7	NA	NA	54.90	7 days Aerobic exercises + inspiratory muscle training
			U	50	64.60	37	NA	45	5	NA	NA	56.00	No preoperative exercise
Sebio 2017 (44)	NCT01963923	Spain	E	10	70.90	0	69.20	NA	NA	NA	NA	90.00	3 weeks, high-intensity training+ resistance training+ inspiratory muscle training
			U	12	69.40	3	87.60	NA	NA	NA	NA	91.67	No preoperative exercise
Bhatia 2019 (45)	NCT01258478	Switzerland	E	74	64.00	NA	86.00	NA	NA	NA	NA	55.00	2–3 weeks, high-intensity interval training
			U	77	64.00	NA	88.00	NA	NA	NA	NA	65.00	no preoperative exercise
Lai 2019 (46)	ChiCTR1800014512	China	E	34	64.20	9	NA	NA	NA	NA	NA	52.94	1 week, aerobic exercises + inspiratory muscle training
			U	34	63.40	11	NA	NA	NA	NA	NA	50.00	no preoperative exercise
Laurent 2020 (12)	2012-A00189-34	France	E	14	64.00	NA	93.00	7	5	NA	NA	64.00	3 weeks, respiratory muscle endurance training + usual chest physical therapy
			U	12	62.00	NA	90.00	7	2	NA	NA	75.00	No preoperative exercise
Liu 2020 (28)	NCT03068507	China	E	37	56.20	3	NA	33	4	33	4	32.00	2 weeks, aerobic and resistance exercises + inspiratory muscle training
			U	36	56.20	2	NA	32	4	32	4	31.00	No preoperative exercise
Patel 2023 (47)	NCT03689634	Canada	E	45	65.53	14	91.69	NA	NA	0	45	31.11	3–4 weeks, aerobic exercises + inspiratory muscle training
			U	50	68.78	17	86.50	NA	NA	0	50	52.00	No preoperative exercise
Zhou 2024 (48)	ChiCTR2200059753	China	E	51	57.00	9	NA	41	3	23	28	37.70	2 weeks, aerobic training + high-intensity interval training
			U	50	56.00	13	NA	42	1	24	26	42.00	No preoperative exercise

NA, not available; E, the preoperative exercise training group; U, the no preoperative exercise group.

### 3.3 Risk of bias

**Figure 2:**
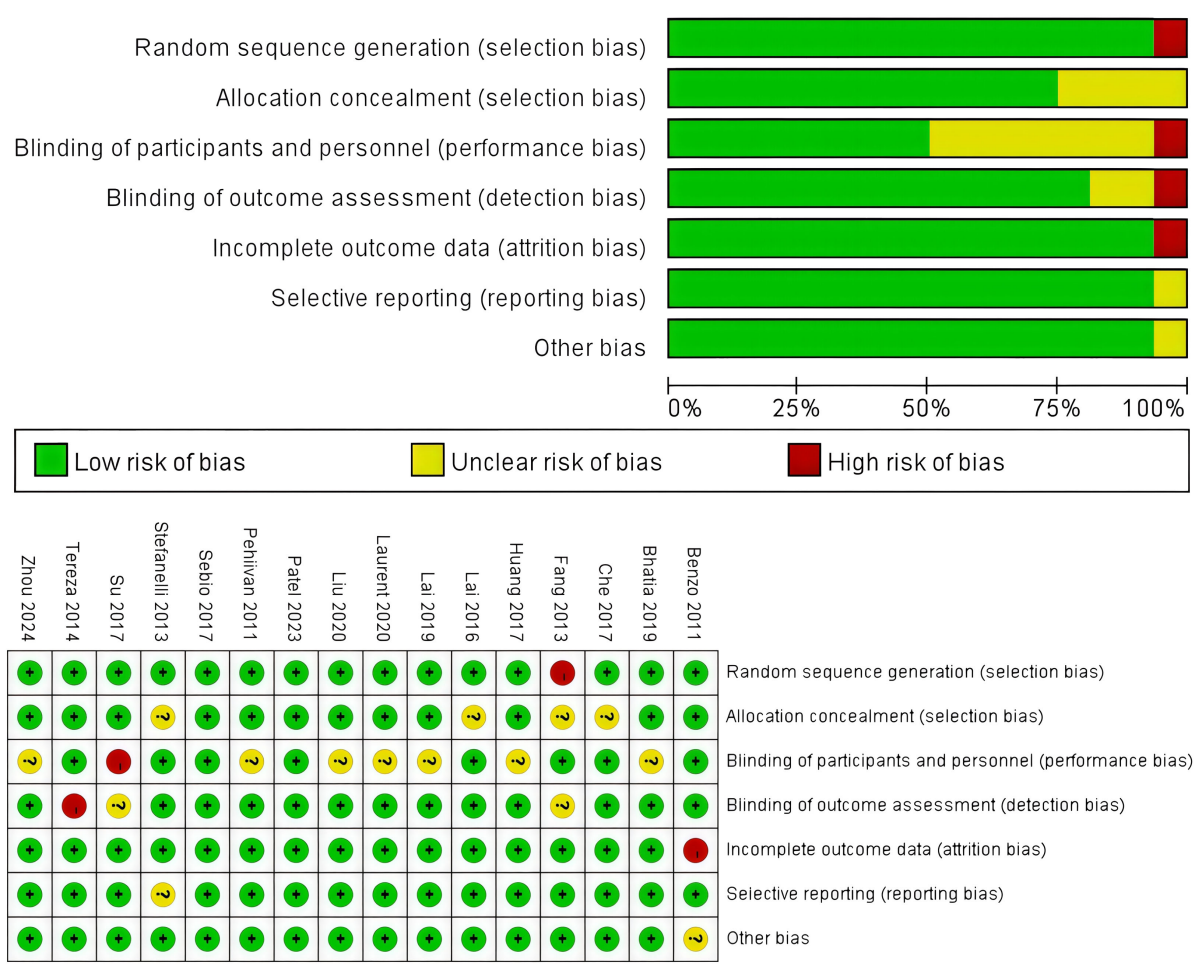
Risk of bias assessment for the RCTs.

The assessment of the risk of bias is summarized in **Figure 2**, and all these 16 RCTs were of high quality. To be more specific, an adequate randomized sequence was reported in 11 RCTs, appropriate allocation concealment was generated in 8 RCTs, the blinding of participants was clear in 12 RCTs, the blinding of outcome assessors was generated in 15 RCTs, outcome data were complete in 15 RCTs, 15 RCTs had no selective reporting, and 15 RCTs had no other bias.

### 3.4 Clinical outcomes

**Table 2** presents the findings of the meta-analysis for all clinical outcomes.

**Table 2:** Results of the meta-analysis.

Outcomes	No. of studies	Sample size		Heterogeneity		Overall effect size	95% CI of overall effect	P value
		Exercise training	no exercise	I^2^(%)	P value			
Postoperative complication	15	503	507	0	0.72	OR = 0.33	0.24 ~ 0.46	< 0.0001
Postoperative length of hospital stay (days)	14	452	457	72	<0.0001	WMD = -2.26	-3.11 ~ -1.40	< 0.0001
FEV1 (L)	6	183	180	87	<0.0001	WMD = 0.18	-0.16 ~ 0.52	0.30
FVC (L)	4	139	138	0	0.97	WMD = 0.10	-0.07 ~ 0.27	0.25
FEV_1_% of predicted norm values	5	113	110	0	0.53	WMD = 6.70	5.30 ~ 8.10	< 0.0001
FVC% of predicted norm values	3	79	78	0	0.51	WMD = 3.07	1.90 ~ 4.23	< 0.0001
PEF (L/min)	3	148	146	8	0.35	WMD = 36.69	12.44 ~ 60.93	0.003
DLCO (mL/min/mmHg)	4	140	140	15	0.32	WMD = 0.88	-0.18 ~ 1.93	0.10
6MWD (m)	7	298	299	86	<0.0001	WMD = 25.55	-18.91 ~ 70.01	0.26
VO_2peak_ (mL/kg/minute)	3	108	109	3	0.36	WMD = 3.29	2.41 ~ 4.17	< 0.0001
Severe postoperative complications (Clavien- Dindo score ≥2)	5	212	210	0	0.76	OR = 0.35	0.21 ~ 0.56	< 0.0001
Postoperative 30-day mortality	8	308	309	0	0.78	OR = 0.46	0.13 ~ 1.67	0.24
Postoperative chest tube drainage time (days)	5	117	118	54	0.07	WMD = -1.65	-3.31 ~ 0.02	0.05
Postintervention dyspnoea	3	131	130	0	0.72	WMD = -0.33	-0.61 ~ -0.04	0.02
Postintervention HRQoL	3	141	140	0	0.92	WMD = 2.28	-0.73 ~ 5.29	0.14

#### 3.4.1 Primary outcomes

##### 3.4.1.1 Postoperative complication

**Figure 3:**
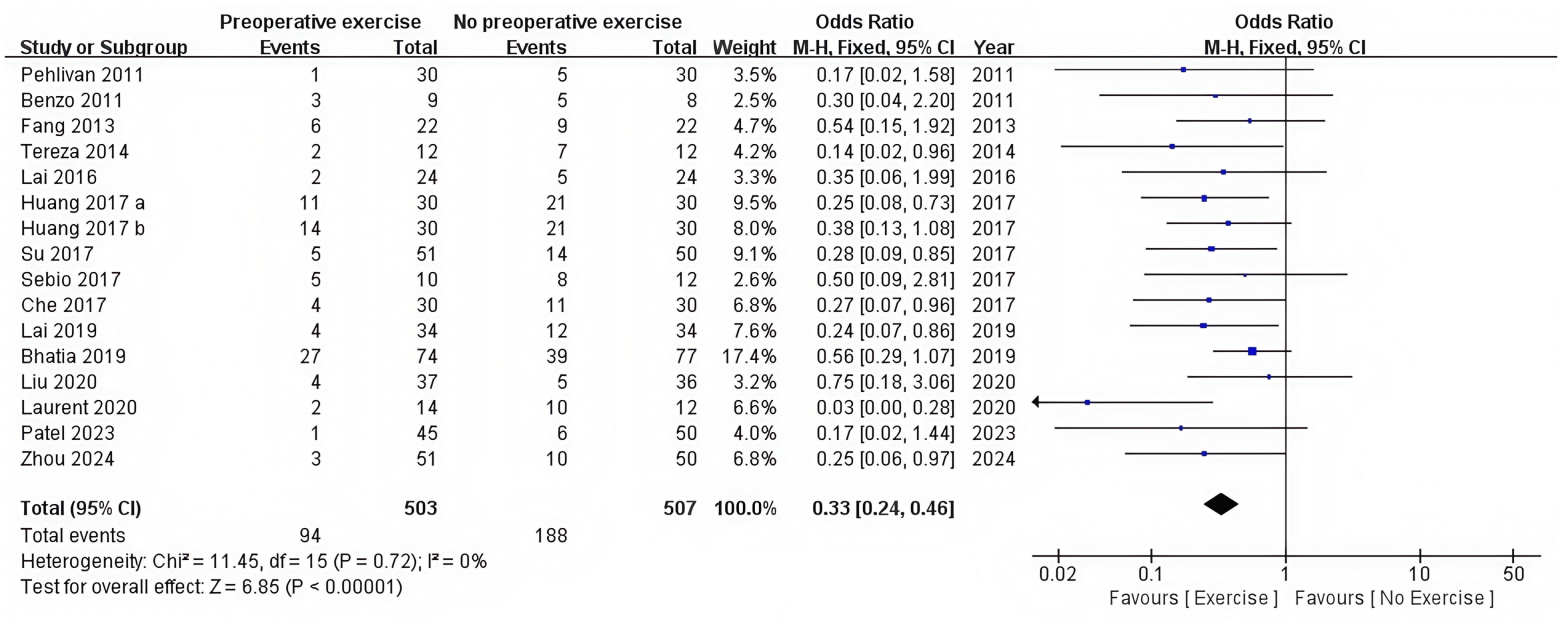
Forest plot of the meta-analysis for postoperative complications.

There were 15 RCTs who reported postoperative complications (12, 28, 36–48). Preoperative exercise training significantly reduced the postoperative complications compared with no preoperative exercise (OR = 0.33, 95%CI: 0.24 to 0.46, P < 0.0001) (**Table 2**, **Figure 3**).

##### 3.4.1.2 Postoperative length of hospital stay (days)

**Figure 4:**
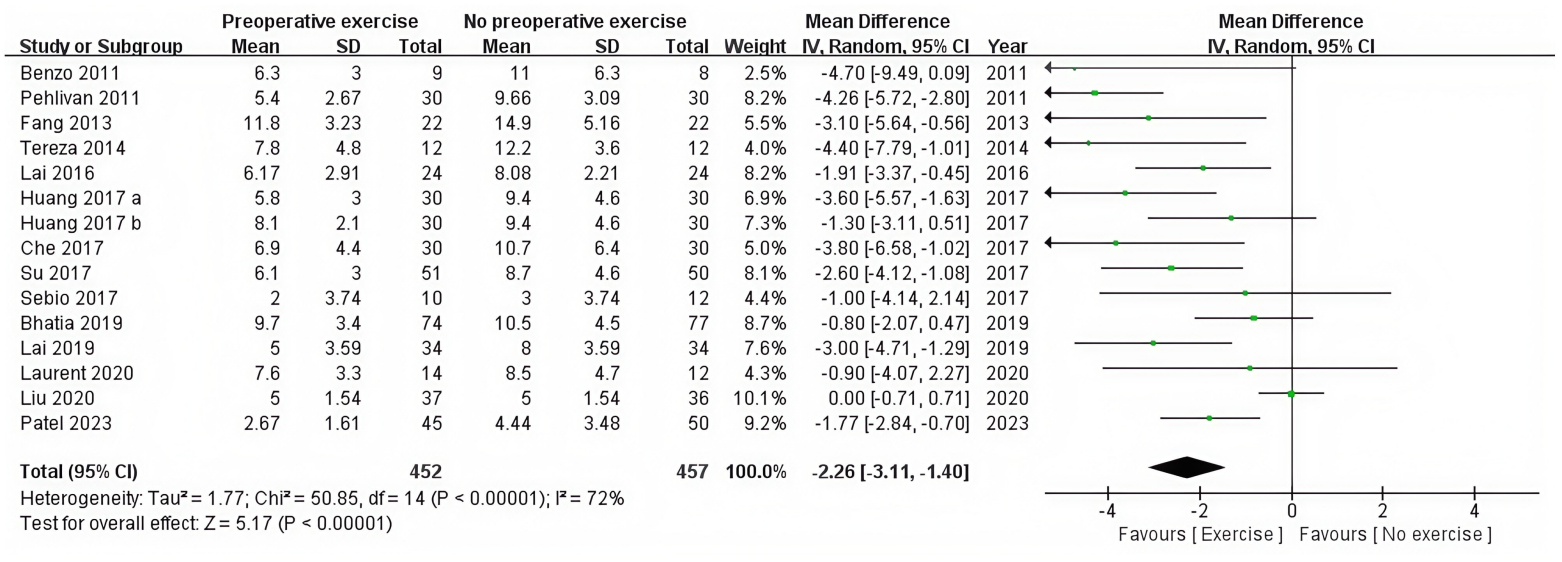
Forest plot of the meta-analysis for postoperative length of hospital stay.

The postoperative hospital stay was recorded in 14 RCTs (12, 28, 36–47). The statistical analysis revealed that preoperative exercise training resulted in a significantly shorter hospital stay compared with usual care (95%CI: −3.11 to −1.40, P < 0.0001) (**Table 2**, **Figure 4**).

#### 3.4.2 Secondary outcomes

##### 3.4.2.1 Post-intervention pulmonary function

Six RCTs compared post-intervention FEV_1_ between preoperative exercise training and no preoperative exercise (12, 28, 37, 39, 41, 42). Preoperative exercise training and no preoperative exercise did not show any statistically significant change (95%CI: −0.16 to 0.52, P = 0.30) (**Table 2**, **Supplementary Figure 1**). Four RCTs investigated the difference in FVC between preoperative exercise training and no preoperative exercise (28, 39, 41, 42). There was no statistically significant change between preoperative exercise training and no preoperative exercise (95%CI: −0.07 to 0.27, P = 0.25) (**Table 2**, **Supplementary Figure 2**). Five RCTs were conducted to compare the FEV_1_% of predicted norm values between preoperative exercise training and no preoperative exercise (12, 28, 29, 37, 39). Preoperative exercise training produced a significantly greater improvement in FEV_1_% of predicted norm values compared with no preoperative exercise (95%CI: 5.30 to 8.10, P < 0.0001) (**Table 2**, **Supplementary Figure 3**). Three RCTs reported the FVC% of predicted norm values for preoperative exercise training and no preoperative exercise (28, 37, 39). Preoperative exercise training significantly enhanced the FVC% of predicted norm values compared with no preoperative exercise (95%CI: 1.90 to 4.23, P < 0.0001) (**Table 2**, **Supplementary Figure 4**). Three RCTs compared PEF between preoperative exercise training and no preoperative exercise (28, 41, 43). Significant disparities existed between preoperative exercise training and no preoperative exercise. The preoperative exercise training significantly enhanced the PEF compared with no preoperative exercise (95%CI: 12.44 to 60.93, P = 0.003) (**Table 2**, **Supplementary Figure 5**). A total of four RCTs documented differences in DLCO between preoperative exercise training and no preoperative exercise (29, 37, 41, 42), and no statistically significant difference was seen between the two groups (95%CI: −0.18 to 1.93, P = 0.10) (**Table 2**, **Supplementary Figure 6**).

##### 3.4.2.2 Post-intervention exercise capacity

Seven RCTs examined the impact of the preoperative exercise training on exercise capacity using the 6MWD text compared with no preoperative exercise (28, 39, 41–43, 45, 46). Preoperative exercise training and no preoperative exercise did not show any statistically significant difference (95%CI: −18.91 to 70.01, P = 0.26) (**Table 2**, **Supplementary Figure 7**). Three RCTs reported post-intervention VO_2peak_ as their measure of exercise capacity (12, 29, 45). Significant disparities existed between preoperative exercise training and no preoperative exercise. Preoperative exercise training increased post-intervention exercise capacity measured by VO_2peak_ (95%CI: 2.41 to 4.17, P < 0.0001) (**Table 2**, **Supplementary Figure 8**).

##### 3.4.2.3 Severe postoperative complications

Five RCTs reported severe postoperative complications (28, 41–43, 46). Preoperative exercise training substantially decreased severe postoperative complications (OR = 0.35, 95%CI: 0.21 to 0.56, P < 0.0001) (**Table 2**, **Supplementary Figure 9**).

##### 3.4.2.4 Postoperative 30-day mortality

Eight RCTs evaluated postoperative 30-day mortality (28, 37, 38, 41, 42, 45, 46, 48). Preoperative exercise training and no preoperative exercise exhibited no statistically significant difference (OR = 0.46, 95%CI: 0.13 to 1.67, P = 0.24) (**Table 2**, **Supplementary Figure 10**).

##### 3.4.2.5 Postoperative chest tube drainage time (days)

Postoperative chest tube drainage time was reported in five RCTs (12, 28, 36, 39, 47). No statistically significant difference was seen between preoperative exercise training and no preoperative exercise (95%CI: −3.31 to 0.02, P = 0.05) (**Table 2**, **Supplementary Figure 11**).

##### 3.4.2.6 Post-intervention dyspnea

Three RCTs documented post-intervention dyspnea on exertion as judged by the BORG scale (29, 41, 43). Preoperative exercise training significantly reduced post-intervention dyspnea compared with no preoperative exercise (95%CI: -0.61 to 0.04, P = 0.02) (**Table 2**, **Supplementary Figure 12**).

##### 3.4.2.7 Post-intervention HRQoL

Three RCTs evaluated post-intervention HRQoL (41–43). There was no statistically significant difference between preoperative exercise training and no preoperative exercise (95%CI: −0.73 to 5.29, P = 0.14) (**Table 2**, **Supplementary Figure 13**).

### 3.5 Publication bias

**Figure 5:**
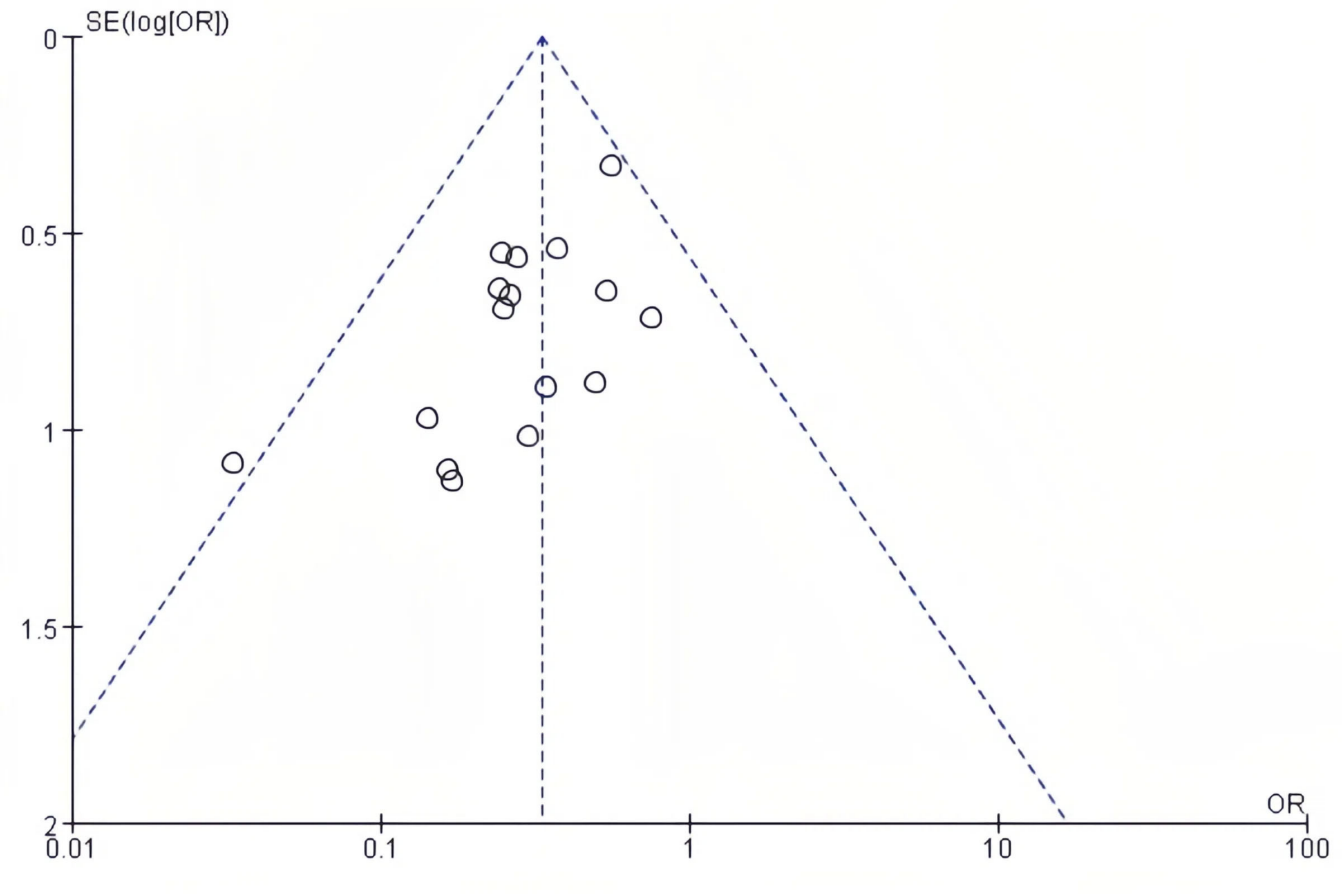
Funnel plot in relation to postoperative complications.

**Figure 6:**
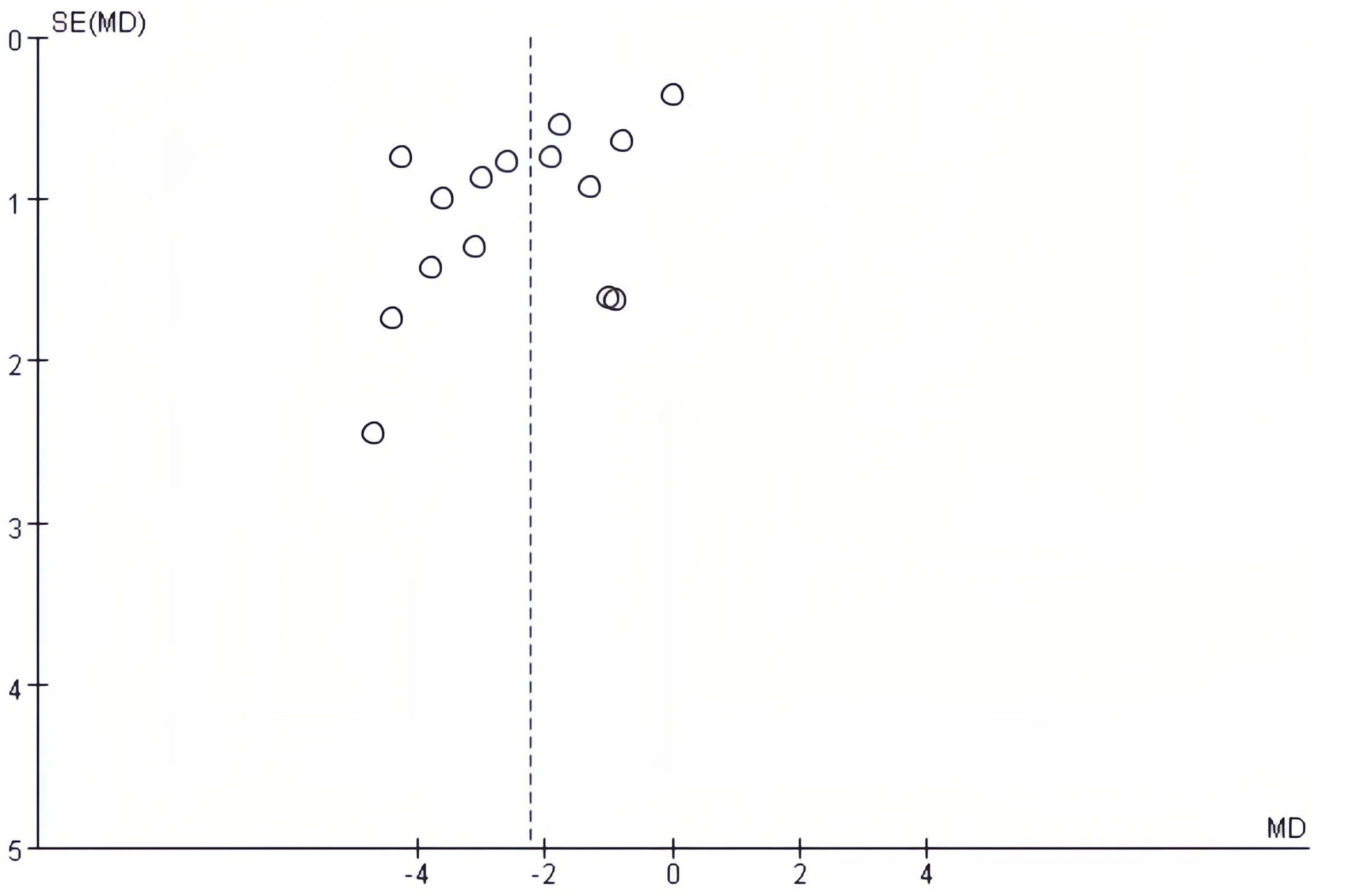
Funnel plot in relation to postoperative length of hospital stay.

The publication bias on postoperative complications and postoperative length of hospital stay was evaluated using funnel plots. There was no notable publication bias detected in the bilaterally symmetrical funnel plots regarding postoperative complications (**Figure 5**) or postoperative length of hospital stay (**Figure 6**).

## 4 Discussion

This updated meta-analysis included 16 RCTs of high quality and assessed the clinical outcomes of patients with lung cancer who received preoperative exercise training. The results of this meta-analysis revealed that preoperative exercise training reduced postoperative complications and decreased postoperative length of hospital stay, which was similar to prior studies (23, 24, 49–52). Preoperative exercise training reduces hypermetabolic, stress, and inflammatory symptoms (53). Practicing deep breathing, coughing, and incentive spirometry before surgery improved lung function and reduced postoperative pneumonia and atelectasis patients (54). Preoperative exercise training can improve inspiratory muscle endurance, exercise capabilities, cardiac output, and muscle oxygen extraction, lowering postoperative complications in the exercise training group. This may boost exercise resistance and aerobic capacity, improving health before surgery and recuperation thereafter (43, 46). Breathing training increases respiratory muscle function, coughing, expectoration, and sputum excretion post-surgery, lowering lung infections and atelectasis (55, 56).

Several pathways have been proposed, which include the modulation of metabolic and sex-steroid hormone levels, enhancement of immune surveillance, reduction of systemic inflammation, and attenuation of oxidative damage through the induction of antioxidant responses to exercise-induced transient oxidative stress, although little evidence substantiates these hypotheses (56–60). The decrease in postoperative complications due to preoperative exercise training likely led to the reduction in postoperative length of hospital stay.

Our findings indicated that preoperative exercise training enhanced post-intervention exercise capacity measured by VO_2peak_ more effectively than no preoperative exercise. Physical deconditioning significantly increases the risk for surgical patients, with low VO_2peak_ serving as an indicator of perioperative mortality and cardiopulmonary complications (61, 62). The VO_2peak_ indicates the comprehensive capacity of the pulmonary, circulatory, and autonomic nervous systems to optimally supply oxygen to the active skeletal muscles (63). Recent data have underscored the power of exercise training to elicit a protective cardiovascular phenotype while improving oxygen extraction in skeletal muscle through increased capillary density and mitochondrial oxidative capacity (64, 65). During maximal activity, the elevated cardiac output, along with enhanced oxygen extraction by the working muscles, leads to an increased VO_2peak_ (66).

Our results demonstrated that preoperative exercise training enhanced preoperative pulmonary function regarding FEV_1_% of predicted norm values, FVC% of predicted norm values, PEF, and decreased preoperative dyspnea. The evidence regarding the impact of preoperative exercise training on lung function was highly equivocal due to the limited number of RCTs that have documented preoperative lung function metrics. The increased occurrence of postoperative complications in the elderly may not be solely attributable to age, but rather to more advanced chronic obstructive pulmonary disease, despite all patients exhibiting similar FEV_1_ levels (67). It was suggested that personalized preoperative exercise training should be obligatory for patients with chronic obstructive pulmonary disease, especially for symptomatic individuals with an FEV_1_ below 50% of the predicted value, and recommended for symptomatic or exercise-limited patients with an FEV_1_ exceeding 50% of the predicted value (68–70). The augmentation of the PEF signifies an improved clearance capacity of endotracheal hypersecretion in the intervention group, suggesting a potential reduction in the postoperative pulmonary complications (PPCs) rate (42). In recent years, several studies have linked PEF to surgical complications, mortality, and the ability to cough and expectorate, which can be used as an index to predict surgery prognosis (40). Preoperative exercise may play a potential role in rendering physiologically inoperable patients operable. Numerous resectable malignancies manifest in patients with impaired lung function, typically attributable to tobacco use, COPD, and/or atherosclerotic vascular disease as underlying comorbidities. This cohort of patients has an elevated risk of surgical complications and may be deemed inoperable (71, 72). The results of this meta-analysis indicated that preoperative exercise training could enhance preoperative pulmonary function. Therefore, it seems reasonable to assume that the patients in high risk of complications and mortality, considered inoperable due to lung function impairment, might be operated after preoperative exercise training. Preliminary findings suggested that preoperative exercise training markedly enhanced cardiopulmonary fitness in low-fit older persons undergoing lobectomy (29). Further evaluation in bigger cohorts and among individuals with highest postoperative risk is necessary.

To our knowledge, this updated meta-analysis included the largest number of RCTs comparing outcomes of preoperative exercise training versus no preoperative exercise for patients with lung cancer who were about to undergo lung resection, which could result in relatively robust conclusions. Nonetheless, we recognize the potential limitations of our study. First of all, the sample size of the included trials were relatively small, and only 16 RCTs were included due to our strict inclusion and exclusion criteria. The statistical results of partial clinical outcomes were difficult to reflect the difference between the two groups due to the relatively small sample size. Second, since the short follow-up periods of the included RCTs, we were unable to analyze long-term outcomes, such as 1-year postoperative mortality. Third, we were unable to manage confounding variables, including varying inclusion criteria, population disparities, and differing intervention of preoperative exercise training. These variables, particularly regarding the variability in exercise interventions and patient populations, may lead to significant heterogeneity. Heterogeneity in exercise interventions, made direct comparisons across studies challenging. Methodological weaknesses or conflicts of interest in included studies might lead to potential selection bias. Fourth, the absence of a gray literature search may contribute to publication bias. Furthermore, certain effects might be overestimated, particularly improvements in VO2peak over short-term interventions. VO2peak thresholds were limited by not using different thresholds for men and women. We failed to resolve this issue because the original literature did not provide data on gender subgroups. In addition, a subject that could provide very interesting information is whether preoperative exercise training improves final outcomes in patients undergoing minimally invasive surgery approaches. However, most of these RCTs did not disaggregate outcome data for patients categorized by type of surgery, which prevented further subgroup analysis regarding minimally invasive procedures. Therefore, more clinical outcomes reported by well-designed RCTs with longer follow-up periods are necessary to further confirm the advantage of preoperative exercise training.

In summary, this meta-analysis indicated that preoperative exercise training was advantageous for lung cancer patients undergoing lung resection, as it could reduce postoperative complications and length of hospital stay, while enhancing post-intervention pulmonary function and exercise capacity.

## Data Availability

The datasets presented in this study can be found in online repositories. The names of the repository/repositories and accession number(s) can be found in the article/**Supplementary Material**.
