# Supplementary material for: Postoperative outcomes of preoperative exercise training in patients with operable non-small cell lung cancer: a systematic review and meta-analysis
Source: Front Oncol. 2025 Sep 12;15:1563478. doi: 10.3389/fonc.2025.1563478 (PMC12463627; doi:10.3389/fonc.2025.1563478)
Supplement: Supplementary file 1 [file DataSheet1.pdf]

PubMed	
No.	Query
1.	(((Lung Cancer[Title/Abstract]) OR (Carcinoma, Non Small Cell Lung[Title/Abstract])) OR (Carcinomas, Non-Small-Cell Lung[Title/Abstract])) OR (Carcinomas, Non Small Cell Lung[Title/Abstract])) OR (Lung Carcinoma, Non-Small-Cell[Title/Abstract])) OR (Lung Carcinomas, Non-Small-Cell[Title/Abstract])) OR (Non-Small-Cell Lung Carcinomas[Title/Abstract])) OR (Non-Small-Cell Lung Carcinoma[Title/Abstract])) OR (Non Small Cell Lung Carcinoma[Title/Abstract])) OR (Non Small Cell Lung Carcinomas[Title/Abstract])) OR (Carcinoma, Non-Small Cell Lung[Title/Abstract])) OR (Carcinomas, Non-Small Cell Lung[Title/Abstract])) OR (Non-Small Cell Lung Carcinoma[Title/Abstract])) OR (Non-Small Cell Lung Carcinomas[Title/Abstract])) OR (Nonsmall Cell Lung Carcinoma[Title/Abstract])) OR (Nonsmall Cell Lung Carcinomas[Title/Abstract])) OR (Non-Small Cell Lung Cancer[Title/Abstract])) OR (Non-Small Cell Lung Cancers[Title/Abstract])) OR (Nonsmall Cell Lung Cancer[Title/Abstract])) OR (Nonsmall Cell Lung Cancers[Title/Abstract])) OR (Cancer, Non-Small-Cell Lung[Title/Abstract])) OR (Cancer, Non Small Cell Lung[Title/Abstract])) OR (Cancers, Non-Small-Cell Lung[Title/Abstract])) OR (Cancers, Non Small Cell Lung[Title/Abstract])) OR (Lung Cancer, Non-Small-Cell[Title/Abstract])) OR (Lung Cancers, Non-Small-Cell[Title/Abstract])) OR (Non-Small-Cell Lung Cancers[Title/Abstract])) OR (Non-Small-Cell Lung Cancer[Title/Abstract])) OR (Non Small Cell Lung Cancer[Title/Abstract])) OR (Non Small Cell Lung Cancers[Title/Abstract])) OR (Cancer, Non-Small Cell Lung[Title/Abstract])) OR (Cancers, Non-Small Cell Lung[Title/Abstract])) OR (Neoplasm, Non-Small-Cell Lung[Title/Abstract])) OR (Neoplasm, Non Small Cell Lung[Title/Abstract])) OR (Neoplasms, Non-Small-Cell Lung[Title/Abstract])) OR (Neoplasms, Non Small Cell Lung[Title/Abstract])) OR (Lung Neoplasm, Non-Small-Cell[Title/Abstract])) OR (Lung Neoplasms, Non-Small-Cell[Title/Abstract])) OR (Non-Small-Cell Lung Neoplasms[Title/Abstract])) OR (Non-Small-Cell Lung Neoplasm[Title/Abstract])) OR (Non Small Cell Lung Neoplasm[Title/Abstract])) OR (Non Small Cell Lung Neoplasms[Title/Abstract])) OR (Neoplasm, Non-Small Cell Lung[Title/Abstract])) OR (Neoplasms, Non-Small Cell Lung[Title/Abstract])) OR (Non-Small Cell Lung Neoplasm[Title/Abstract])) OR (Non-Small Cell Lung Neoplasms[Title/Abstract])) OR (Nonsmall Cell Lung Neoplasm[Title/Abstract])) OR (Nonsmall Cell Lung Neoplasms[Title/Abstract])
2.	((((((((((((((((((((((((((((((Preoperative Exercise[Title/Abstract]) OR (Exercise, Preoperative[Title/Abstract])) OR (Preoperative Exercises[Title/Abstract])) OR (Exercises, Preoperative[Title/Abstract])) OR (Exercise, Preoperative[Title/Abstract])) OR (Pre-operative Conditioning[Title/Abstract])) OR (Conditioning, Pre-operative[Title/Abstract])) OR (Pre operative Conditioning[Title/Abstract])) OR (Pre-operative Conditionings[Title/Abstract])) OR (Conditionings, Pre-operative[Title/Abstract])) OR (Pre-operative Rehabilitation[Title/Abstract])) OR (Pre operative Rehabilitation[Title/Abstract])) OR (Pre-operative Rehabilitations[Title/Abstract])) OR (Rehabilitations Pre-operative[Title/Abstract])) OR (Rehabilitation, Pre-operative[Title/Abstract])) OR (Preoperative Rehabilitation[Title/Abstract])) OR (Preoperative Conditioning[Title/Abstract]))

	OR (Preoperative Conditionings[Title/Abstract])) OR (Preoperative Rehabilitations[Title/Abstract])) OR (Rehabilitation, Preoperative[Title/Abstract])) OR (Rehabilitations, Preoperative[Title/Abstract])) OR (Conditioning, Preoperative[Title/Abstract])) OR (Conditionings, Preoperative[Title/Abstract])) OR (Pre-operative Exercise[Title/Abstract])) OR (Exercise, Pre-operative[Title/Abstract])) OR (Exercises, Pre-operative[Title/Abstract])) OR (Pre operative Exercise[Title/Abstract])) OR (Pre operative Exercises[Title/Abstract])) OR (Pre-operative Exercises[Title/Abstract])) OR (Pre-operative Exercise[Title/Abstract])) OR (Prehabilitation[Title/Abstract])
3.	(((((((((((((((Randomized Controlled Trial[Title/Abstract]) OR (Randomized Controlled Trials[Title/Abstract])) OR (Clinical Trials, Randomized[Title/Abstract])) OR (Clinical Trial, Randomized[Title/Abstract])) OR (Trials, Randomized Clinical[Title/Abstract])) OR (Trial, Randomized Clinical[Title/Abstract])) OR (Controlled Clinical Trials, Randomized[Title/Abstract])) OR (Controlled Clinical Trial, Randomized[Title/Abstract])) OR (controlled clinical trial[Title/Abstract])) OR (controlled clinical trials[Title/Abstract])) OR (Randomized[Title/Abstract])) OR (placebo[Title/Abstract])) OR (clinical trials as topic[Title/Abstract])) OR (clinical trial as topic[Title/Abstract])) OR (randomly[Title/Abstract])) OR (Trial[Title/Abstract])) OR (Trials[Title/Abstract])
4.	((((((((((((High-Intensity Interval Training[Title/Abstract]) OR (High Intensity Interval Training[Title/Abstract])) OR (High-Intensity Interval Trainings[Title/Abstract])) OR (High Intensity Interval Trainings[Title/Abstract])) OR (Interval Training, High-Intensity[Title/Abstract])) OR (Interval Trainings, High-Intensity[Title/Abstract])) OR (Training, High-Intensity Interval[Title/Abstract])) OR (Trainings, High-Intensity Interval[Title/Abstract])) OR (High-Intensity Intermittent Exercise[Title/Abstract])) OR (Exercise, High-Intensity Intermittent[Title/Abstract])) OR (Exercises, High-Intensity Intermittent[Title/Abstract])) OR (High-Intensity Intermittent Exercises[Title/Abstract])) OR (Sprint Interval Training[Title/Abstract])) OR (Sprint Interval Trainings[Title/Abstract])
5	((((((((((((((((((((((((((Resistance Training[Title/Abstract]) OR (Training, Resistance[Title/Abstract])) OR (Strength Training[Title/Abstract])) OR (Training, Strength[Title/Abstract])) OR (Weight-Lifting Strengthening Program[Title/Abstract])) OR (Strengthening Program, Weight-Lifting[Title/Abstract])) OR (Strengthening Programs, Weight-Lifting[Title/Abstract])) OR (Weight Lifting Strengthening Program[Title/Abstract])) OR (Weight Lifting Strengthening Programs[Title/Abstract])) OR (Weight-Lifting Strengthening Programs[Title/Abstract])) OR (Weight-Lifting Exercise Program[Title/Abstract])) OR (Exercise Program, Weight-Lifting[Title/Abstract])) OR (Exercise Programs, Weight-Lifting[Title/Abstract])) OR (Weight Lifting Exercise Program[Title/Abstract])) OR (Weight Lifting Exercise Programs[Title/Abstract])) OR (Weight-Lifting Exercise Programs[Title/Abstract])) OR (Weight-Bearing Strengthening Program[Title/Abstract])) OR (Strengthening Program, Weight-Bearing[Title/Abstract])) OR (Strengthening Programs, Weight-Bearing[Title/Abstract])) OR (Weight Bearing Strengthening Program[Title/Abstract])) OR (Weight Bearing Strengthening Programs[Title/Abstract])) OR (Weight-Bearing Strengthening Programs[Title/Abstract])) OR (Weight-Bearing Exercise Program[Title/Abstract])) OR (Weight-Bearing Exercise Programs[Title/Abstract])) OR (Exercise Program, Weight-Bearing[Title/Abstract])) OR (Exercise Programs, Weight-Bearing[Title/Abstract])) OR (Weight Bearing Exercise

	Program[Title/Abstract])) OR (Weight Bearing Exercise Programs[Title/Abstract])
6.	((((Aerobic training[Title/Abstract]) OR (Exercise, Aerobic[Title/Abstract])) OR (Aerobic Exercise[Title/Abstract])) OR (Aerobic Exercises[Title/Abstract])) OR (Exercises, Aerobic[Title/Abstract])
7.	((((((Breathing Exercises[Title/Abstract]) OR (Exercises, Breathing[Title/Abstract])) OR (Exercise, Breathing[Title/Abstract])) OR (Breathing Exercise[Title/Abstract])) OR (Respiratory Muscle Training[Title/Abstract])) OR (Muscle Training, Respiratory[Title/Abstract])) OR (Training, Respiratory Muscle[Title/Abstract])
8.	2 OR 4 OR 5 OR 6 OR 7
9.	8 AND 1
10.	1 AND 3
	9 AND 10

Web of science	
No.	Query
1.	(((TS=(Lung Cancer)) OR TS=(Carcinoma, Non Small Cell Lung)) OR TS=(Carcinomas, Non-Small-Cell Lung)) OR TS=(Carcinomas, Non Small Cell Lung)) OR TS=(Lung Carcinoma, Non-Small-Cell)) OR TS=(Lung Carcinomas, Non-Small-Cell)) OR TS=(Non-Small-Cell Lung Carcinomas)) OR TS=(Non-Small-Cell Lung Carcinoma)) OR TS=(Non Small Cell Lung Carcinoma)) OR TS=(Non Small Cell Lung Carcinomas)) OR TS=(Carcinoma, Non-Small Cell Lung)) OR TS=(Carcinomas, Non-Small Cell Lung)) OR TS=(Non-Small Cell Lung Carcinoma)) OR TS=(Non-Small Cell Lung Carcinomas)) OR TS=(Nonsmall Cell Lung Carcinoma)) OR TS=(Nonsmall Cell Lung Carcinomas)) OR TS=(Non-Small Cell Lung Cancer)) OR TS=(Non-Small Cell Lung Cancers)) OR TS=(Nonsmall Cell Lung Cancer)) OR TS=(Nonsmall Cell Lung Cancers)) OR TS=(Cancer, Non-Small-Cell Lung)) OR TS=(Cancer, Non Small Cell Lung)) OR TS=(Cancers, Non-Small-Cell Lung)) OR TS=(Cancers, Non Small Cell Lung)) OR TS=(Lung Cancer, Non-Small-Cell)) OR TS=(Lung Cancers, Non-Small-Cell)) OR TS=(Non-Small-Cell Lung Cancers)) OR TS=(Non-Small-Cell Lung Cancer)) OR TS=(Non Small Cell Lung Cancer)) OR TS=(Non Small Cell Lung Cancers)) OR TS=(Cancer, Non-Small Cell Lung)) OR TS=(Cancers, Non-Small Cell Lung)) OR TS=(Neoplasm, Non-Small-Cell Lung)) OR TS=(Neoplasm, Non Small Cell Lung)) OR TS=(Neoplasms, Non-Small-Cell Lung)) OR TS=(Neoplasms, Non Small Cell Lung)) OR TS=(Lung Neoplasm, Non-Small-Cell)) OR TS=(Lung Neoplasms, Non-Small-Cell)) OR TS=(Non-Small-Cell Lung Neoplasms)) OR TS=(Non-Small-Cell Lung Neoplasm)) OR TS=(Non Small Cell Lung Neoplasm)) OR TS=(Non Small Cell Lung Neoplasms)) OR TS=(Neoplasm, Non-Small Cell Lung)) OR TS=(Neoplasms, Non-Small Cell Lung)) OR TS=(Non-Small Cell Lung Neoplasm)) OR TS=(Non-Small Cell Lung Neoplasms)) OR TS=(Nonsmall Cell Lung Neoplasm)) OR TS=(Nonsmall Cell Lung Neoplasms)

2.	((((((((((((((((((((((((((((((TS=(Preoperative Exercise)) OR TS=(Exercise, Preoperative)) OR TS=(Preoperative Exercises)) OR TS=(Exercises, Preoperative)) OR TS=(Exercise, Preoperative)) OR TS=(Pre-operative Conditioning)) OR TS=(Conditioning, Pre-operative)) OR TS=(Pre operative Conditioning)) OR TS=(Pre-operative Conditionings)) OR TS=(Conditionings, Pre-operative)) OR TS=(Pre-operative Rehabilitation)) OR TS=(Pre operative Rehabilitation)) OR TS=(Pre-operative Rehabilitations)) OR TS=(Rehabilitations Pre-operative)) OR TS=(Rehabilitation, Pre-operative)) OR TS=(Preoperative Rehabilitation)) OR TS=(Preoperative Conditioning)) OR TS=(Preoperative Conditionings)) OR TS=(Preoperative Rehabilitations)) OR TS=(Rehabilitation, Preoperative)) OR TS=(Rehabilitations, Preoperative)) OR TS=(Conditioning, Preoperative)) OR TS=(Conditionings, Preoperative)) OR TS=(Pre-operative Exercise)) OR TS=(Exercise, Pre-operative)) OR TS=(Exercises, Pre-operative)) OR TS=(Pre operative Exercise)) OR TS=(Pre operative Exercises)) OR TS=(Pre-operative Exercises)) OR TS=(Pre-operative Exercise)) OR TS=(Prehabilitation)
3.	((((((((((((((((TS=(Randomized Controlled Trial)) OR TS=(Randomized Controlled Trials)) OR TS=(Clinical Trials, Randomized)) OR TS=(Clinical Trial, Randomized)) OR TS=(Trials, Randomized Clinical)) OR TS=(Trial, Randomized Clinical)) OR TS=(Controlled Clinical Trials, Randomized)) OR TS=(Controlled Clinical Trial, Randomized)) OR TS=(controlled clinical trial)) OR TS=(controlled clinical trials)) OR TS=(Randomized)) OR TS=(placebo)) OR TS=(clinical trials as topic)) OR TS=(clinical trial as topic)) OR TS=(randomly)) OR TS=(Trial)) OR TS=(Trials)
4.	(((((((((((((TS=(High-Intensity Interval Training)) OR TS=(High Intensity Interval Training)) OR TS=(High-Intensity Interval Trainings)) OR TS=(High Intensity Interval Trainings)) OR TS=(Interval Training, High-Intensity)) OR TS=(Interval Trainings, High-Intensity)) OR TS=(Training, High-Intensity Interval)) OR TS=(Trainings, High-Intensity Interval)) OR TS=(High-Intensity Intermittent Exercise)) OR TS=(Exercise, High-Intensity Intermittent)) OR TS=(Exercises, High-Intensity Intermittent)) OR TS=(High-Intensity Intermittent Exercises)) OR TS=(Sprint Interval Training)) OR TS=(Sprint Interval Trainings)
5.	(((((((((((((((((((((((((((TS=(Resistance Training)) OR TS=(Training, Resistance)) OR TS=(Strength Training)) OR TS=(Training, Strength)) OR TS=(Weight-Lifting Strengthening Program)) OR TS=(Strengthening Program, Weight-Lifting)) OR TS=(Strengthening Programs, Weight-Lifting)) OR TS=(Weight Lifting Strengthening Program)) OR TS=(Weight Lifting Strengthening Programs)) OR TS=(Weight-Lifting Strengthening Programs)) OR TS=(Weight-Lifting Exercise Program)) OR TS=(Exercise Program, Weight-Lifting)) OR TS=(Exercise Programs, Weight-Lifting)) OR TS=(Weight Lifting Exercise Program)) OR TS=(Weight Lifting Exercise Programs)) OR TS=(Weight-Lifting Exercise Programs)) OR TS=(Weight-Bearing Strengthening Program)) OR TS=(Strengthening Program, Weight-Bearing)) OR TS=(Strengthening Programs, Weight-Bearing)) OR TS=(Weight Bearing Strengthening Program)) OR TS=(Weight Bearing Strengthening Programs)) OR TS=(Weight-Bearing Strengthening Programs)) OR TS=(Weight-Bearing Exercise Program)) OR TS=(Weight-Bearing Exercise Programs)) OR TS=(Exercise Program, Weight-Bearing)) OR TS=(Exercise Programs, Weight-Bearing)) OR TS=(Weight Bearing Exercise Program)) OR TS=(Weight Bearing Exercise Programs)
6.	((((TS=(Aerobic training)) OR TS=(Exercise, Aerobic)) OR TS=(Aerobic Exercise)) OR

	TS=(Aerobic Exercises)) OR TS=(Exercises, Aerobic)
7.	((((((TS=(Breathing Exercises)) OR TS=(Exercises, Breathing)) OR TS=(Exercise, Breathing)) OR TS=(Breathing Exercise)) OR TS=(Respiratory Muscle Training)) OR TS=(Muscle Training, Respiratory)) OR TS=(Training, Respiratory Muscle)
8.	2 OR 4 OR 5 OR 6 OR 7
9.	8 AND 1
10	1 AND 3
	9 AND 10

Embase	
No.	Query
1.	'Lung Cancer'/exp OR 'Carcinoma, Non Small Cell Lung':ab,ti,kw OR 'Carcinomas, Non-Small-Cell Lung':ab,ti,kw OR 'Carcinomas, Non Small Cell Lung':ab,ti,kw OR 'Lung Carcinoma, Non-Small-Cell':ab,ti,kw OR 'Lung Carcinomas, Non-Small-Cell':ab,ti,kw OR 'Non-Small-Cell Lung Carcinomas':ab,ti,kw OR 'Non-Small-Cell Lung Carcinoma':ab,ti,kw OR 'Non Small Cell Lung Carcinoma':ab,ti,kw OR 'Non Small Cell Lung Carcinomas':ab,ti,kw OR 'Carcinoma, Non-Small Cell Lung':ab,ti,kw OR 'Carcinomas, Non-Small Cell Lung':ab,ti,kw OR 'Non-Small Cell Lung Carcinoma':ab,ti,kw OR 'Non-Small Cell Lung Carcinomas':ab,ti,kw OR 'Non-Small Cell Lung Cancer':ab,ti,kw OR 'Non-Small Cell Lung Cancers':ab,ti,kw OR 'Nonsmall Cell Lung Cancer':ab,ti,kw OR 'Nonsmall Cell Lung Cancers':ab,ti,kw OR 'Cancer, Non-Small-Cell Lung':ab,ti,kw OR 'Cancer, Non Small Cell Lung':ab,ti,kw OR 'Cancers, Non-Small-Cell Lung':ab,ti,kw OR 'Cancers, Non Small Cell Lung':ab,ti,kw OR 'Lung Cancer, Non-Small-Cell':ab,ti,kw OR 'Lung Cancers, Non-Small-Cell':ab,ti,kw OR 'Non-Small-Cell Lung Cancers':ab,ti,kw OR 'Non-Small-Cell Lung Cancer':ab,ti,kw OR 'Non Small Cell Lung Cancer':ab,ti,kw OR 'Non Small Cell Lung Cancers':ab,ti,kw OR 'Cancer, Non-Small Cell Lung':ab,ti,kw OR 'Cancers, Non-Small Cell Lung':ab,ti,kw OR 'Non-Small Cell Lung Cancer':ab,ti,kw OR 'Non-Small Cell Lung Cancers':ab,ti,kw OR 'Nonsmall Cell Lung Carcinoma':ab,ti,kw OR 'Nonsmall Cell Lung Carcinomas':ab,ti,kw OR 'Neoplasm, Non-Small-Cell Lung':ab,ti,kw OR 'Neoplasm, Non Small Cell Lung':ab,ti,kw OR 'Neoplasms, Non-Small-Cell Lung':ab,ti,kw OR 'Neoplasms, Non Small Cell Lung':ab,ti,kw OR 'Lung Neoplasm, Non-Small-Cell':ab,ti,kw OR 'Lung Neoplasms, Non-Small-Cell':ab,ti,kw OR 'Non-Small-Cell Lung Neoplasms':ab,ti,kw OR 'Non-Small-Cell Lung Neoplasm':ab,ti,kw OR 'Non Small Cell Lung Neoplasm':ab,ti,kw OR 'Non Small Cell Lung Neoplasms':ab,ti,kw OR 'Neoplasm, Non-Small Cell Lung':ab,ti,kw OR 'Neoplasms, Non-Small Cell Lung':ab,ti,kw OR 'Non-Small Cell Lung Neoplasm':ab,ti,kw OR 'Non-Small Cell Lung Neoplasms':ab,ti,kw OR 'Nonsmall Cell Lung Neoplasm':ab,ti,kw OR 'Nonsmall Cell Lung Neoplasms':ab,ti,kw
2.	'Preoperative Exercise'/exp OR 'Exercise, Preoperative':ab,ti,kw OR 'Preoperative Exercises':ab,ti,kw OR 'Exercises, Preoperative':ab,ti,kw OR 'Exercise, Preoperative':ab,ti,kw OR 'Pre-operative Conditioning':ab,ti,kw OR 'Conditioning, Pre-operative':ab,ti,kw OR 'Pre

	operative Conditioning':ab,ti,kw OR 'Pre-operative Conditionings':ab,ti,kw OR 'Conditionings, Pre-operative':ab,ti,kw OR 'Pre-operative Rehabilitation':ab,ti,kw OR 'Pre operative Rehabilitation':ab,ti,kw OR 'Pre-operative Rehabilitations':ab,ti,kw OR 'Rehabilitations Pre-operative':ab,ti,kw OR 'Rehabilitation, Pre-operative':ab,ti,kw OR 'Preoperative Rehabilitation':ab,ti,kw OR 'Preoperative Conditioning':ab,ti,kw OR 'Preoperative Conditionings':ab,ti,kw OR 'Preoperative Rehabilitations':ab,ti,kw OR 'Rehabilitation, Preoperative':ab,ti,kw OR 'Rehabilitations, Preoperative':ab,ti,kw OR 'Conditioning, Preoperative':ab,ti,kw OR 'Conditionings, Preoperative':ab,ti,kw OR 'Pre-operative Exercise':ab,ti,kw OR 'Exercise, Pre-operative':ab,ti,kw OR 'Exercises, Pre-operative':ab,ti,kw OR 'Pre operative Exercise':ab,ti,kw OR 'Pre operative Exercises':ab,ti,kw OR 'Pre-operative Exercises':ab,ti,kw OR 'Pre-operative Exercise':ab,ti,kw OR 'Prehabilitation':ab,ti,kw
3.	'Randomized Controlled Trial'/exp OR 'Randomized Controlled Trials':ab,ti,kw OR 'Clinical Trials, Randomized':ab,ti,kw OR 'Clinical Trial, Randomized':ab,ti,kw OR 'Trials, Randomized Clinical':ab,ti,kw OR 'Trial, Randomized Clinical':ab,ti,kw OR 'Controlled Clinical Trials, Randomized':ab,ti,kw OR 'Controlled Clinical Trial, Randomized':ab,ti,kw OR 'controlled clinical trial':ab,ti,kw OR 'controlled clinical trials':ab,ti,kw OR 'Randomized':ab,ti,kw OR 'placebo':ab,ti,kw OR 'clinical trials as topic':ab,ti,kw OR 'clinical trial as topic':ab,ti,kw OR 'randomly':ab,ti,kw OR 'Trial':ab,ti,kw OR 'Trials':ab,ti,kw
4.	'High-Intensity Interval Training'/exp OR 'High Intensity Interval Training':ab,ti,kw OR 'High-Intensity Interval Trainings':ab,ti,kw OR 'High Intensity Interval Trainings':ab,ti,kw OR 'Interval Training, High-Intensity':ab,ti,kw OR 'Interval Trainings, High-Intensity':ab,ti,kw OR 'Training, High-Intensity Interval':ab,ti,kw OR 'Trainings, High-Intensity Interval':ab,ti,kw OR 'High-Intensity Intermittent Exercise':ab,ti,kw OR 'Exercise, High-Intensity Intermittent':ab,ti,kw OR 'Exercises, High-Intensity Intermittent':ab,ti,kw OR 'High-Intensity Intermittent Exercises':ab,ti,kw OR 'Sprint Interval Training':ab,ti,kw OR 'Sprint Interval Trainings':ab,ti,kw
5.	'Resistance Training'/exp OR 'Training, Resistance':ab,ti,kw OR 'Strength Training':ab,ti,kw OR 'Training, Strength':ab,ti,kw OR 'Weight-Lifting Strengthening Program':ab,ti,kw OR 'Strengthening Program, Weight-Lifting':ab,ti,kw OR 'Strengthening Programs, Weight-Lifting':ab,ti,kw OR 'Weight Lifting Strengthening Program':ab,ti,kw OR 'Weight Lifting Strengthening Programs':ab,ti,kw OR 'Weight-Lifting Strengthening Programs':ab,ti,kw OR 'Weight-Lifting Exercise Program':ab,ti,kw OR 'Exercise Program, Weight-Lifting':ab,ti,kw OR 'Exercise Programs, Weight-Lifting':ab,ti,kw OR 'Weight Lifting Exercise Program':ab,ti,kw OR 'Weight Lifting Exercise Programs':ab,ti,kw OR 'Weight-Lifting Exercise Programs':ab,ti,kw OR 'Weight-Bearing Strengthening Program':ab,ti,kw OR 'Strengthening Program, Weight-Bearing':ab,ti,kw OR 'Strengthening Programs, Weight-Bearing':ab,ti,kw OR 'Weight Bearing Strengthening Program':ab,ti,kw OR 'Weight Bearing Strengthening Programs':ab,ti,kw OR 'Weight-Bearing Strengthening Programs':ab,ti,kw OR 'Weight-Bearing Exercise Program':ab,ti,kw OR 'Weight-Bearing Exercise Programs':ab,ti,kw OR 'Exercise Program, Weight-Bearing':ab,ti,kw OR 'Exercise Programs, Weight-Bearing':ab,ti,kw OR 'Weight Bearing Exercise Program':ab,ti,kw OR 'Weight Bearing Exercise Programs':ab,ti,kw
6.	'Aerobic training'/exp OR 'Exercise, Aerobic':ab,ti,kw OR 'Aerobic Exercise':ab,ti,kw OR

	‘Aerobic Exercises’:ab,ti,kw OR ‘Exercises, Aerobic’:ab,ti,kw
7.	‘Breathing Exercises’/exp OR ‘Exercises, Breathing’:ab,ti,kw OR ‘Exercise, Breathing’:ab,ti,kw OR ‘Breathing Exercise’:ab,ti,kw OR ‘Respiratory Muscle Training’:ab,ti,kw OR ‘Muscle Training, Respiratory’:ab,ti,kw OR ‘Training, Respiratory Muscle’:ab,ti,kw
8.	2 OR 4 OR 5 OR 6 OR 7
9.	8 AND 1
10.	1 AND 3
	9 AND 10

Cochrance library	
No.	Query
1.	(Lung Cancer OR Carcinoma, Non Small Cell Lung OR Carcinomas, Non-Small-Cell Lung OR Carcinomas, Non Small Cell Lung OR Lung Carcinoma, Non-Small-Cell OR Lung Carcinomas, Non-Small-Cell OR Non-Small-Cell Lung Carcinomas OR Non-Small-Cell Lung Carcinoma OR Non Small Cell Lung Carcinoma OR Non Small Cell Lung Carcinomas OR Carcinoma, Non-Small Cell Lung OR Carcinomas, Non-Small Cell Lung OR Non-Small Cell Lung Carcinoma OR Non-Small Cell Lung Carcinomas OR Non-Small Cell Lung Cancer OR Non-Small Cell Lung Cancers OR Nonsmall Cell Lung Cancer OR Nonsmall Cell Lung Cancers OR Cancer, Non-Small-Cell Lung OR Cancer, Non Small Cell Lung OR Cancers, Non-Small-Cell Lung OR Cancers, Non Small Cell Lung OR Lung Cancer, Non-Small-Cell OR Lung Cancers, Non-Small-Cell OR Non-Small-Cell Lung Cancers OR Non-Small-Cell Lung Cancer OR Non Small Cell Lung Cancer OR Non Small Cell Lung Cancers OR Cancer, Non-Small Cell Lung OR Cancers, Non-Small Cell Lung OR Non-Small Cell Lung Cancer OR Non-Small Cell Lung Cancers OR Nonsmall Cell Lung Carcinoma OR Nonsmall Cell Lung Carcinomas OR Neoplasm, Non-Small-Cell Lung OR Neoplasm, Non Small Cell Lung OR Neoplasms, Non-Small-Cell Lung OR Neoplasms, Non Small Cell Lung OR Lung Neoplasm, Non-Small-Cell OR Lung Neoplasms, Non-Small-Cell OR Non-Small-Cell Lung Neoplasms OR Non-Small-Cell Lung Neoplasm OR Non Small Cell Lung Neoplasm OR Non Small Cell Lung Neoplasms OR Neoplasm, Non-Small Cell Lung OR Neoplasms, Non-Small Cell Lung OR Non-Small Cell Lung Neoplasm OR Non-Small Cell Lung Neoplasms OR Nonsmall Cell Lung Neoplasm OR Nonsmall Cell Lung Neoplasms):ab,ti,kw
2.	(Preoperative Exercise OR Exercise, Preoperative OR Preoperative Exercises OR Exercises, Preoperative OR Exercise, Preoperative OR Pre-operative Conditioning OR Conditioning, Pre-operative OR Pre operative Conditioning OR Pre-operative Conditionings OR Conditionings, Pre-operative OR Pre-operative Rehabilitation OR Pre operative Rehabilitation OR Pre-operative Rehabilitations OR Rehabilitations Pre-operative OR Rehabilitation, Pre-operative OR Preoperative Rehabilitation OR Preoperative Conditioning OR Preoperative Conditionings OR Preoperative Rehabilitations OR Rehabilitation, Preoperative OR Rehabilitations, Preoperative OR Conditioning, Preoperative OR Conditionings, Preoperative OR Pre-operative Exercise OR Exercise, Pre-operative OR Exercises, Pre-operative OR Pre operative Exercise OR Pre operative Exercises OR Pre-operative Exercises OR Pre-operative Exercise OR Prehabilitation):ab,ti,kw

3.	(Randomized Controlled Trial OR Randomized Controlled Trials OR Clinical Trials, Randomized OR Clinical Trial, Randomized OR Trials, Randomized Clinical OR Trial, Randomized Clinical OR Controlled Clinical Trials, Randomized OR Controlled Clinical Trial, Randomized OR controlled clinical trial OR controlled clinical trials OR Randomized OR placebo OR clinical trials as topic OR clinical trial as topic OR randomly OR Trial OR Trials):ab,ti,kw
4.	(High-Intensity Interval Training OR High Intensity Interval Training OR High-Intensity Interval Trainings OR High Intensity Interval Trainings OR Interval Training, High-Intensity OR Interval Trainings, High-Intensity OR Training, High-Intensity Interval OR Trainings, High-Intensity Interval OR High-Intensity Intermittent Exercise OR Exercise, High-Intensity Intermittent OR Exercises, High-Intensity Intermittent OR High-Intensity Intermittent Exercises OR Sprint Interval Training OR Sprint Interval Trainings):ab,ti,kw
5	(Resistance Training OR Training, Resistance OR Strength Training OR Training, Strength OR Weight-Lifting Strengthening Program OR Strengthening Program, Weight-Lifting OR Strengthening Programs, Weight-Lifting OR Weight Lifting Strengthening Program OR Weight Lifting Strengthening Programs OR Weight-Lifting Strengthening Programs OR Weight-Lifting Exercise Program OR Exercise Program, Weight-Lifting OR Exercise Programs, Weight-Lifting OR Weight Lifting Exercise Program OR Weight Lifting Exercise Programs OR Weight-Lifting Exercise Programs OR Weight-Bearing Strengthening Program OR Strengthening Program, Weight-Bearing OR Strengthening Programs, Weight-Bearing OR Weight Bearing Strengthening Program OR Weight Bearing Strengthening Programs OR Weight-Bearing Strengthening Programs OR Weight-Bearing Exercise Program OR Weight-Bearing Exercise Programs OR Exercise Program, Weight-Bearing OR Exercise Programs, Weight-Bearing OR Weight Bearing Exercise Program OR Weight Bearing Exercise Programs):ab,ti,kw
6	(Aerobic training OR Exercise, Aerobic OR Aerobic Exercise OR Aerobic Exercises OR Exercises, Aerobic):ab,ti,kw
7	(Breathing Exercises OR Exercises, Breathing OR Exercise, Breathing OR Breathing Exercise OR Respiratory Muscle Training OR Muscle Training, Respiratory OR Training, Respiratory Muscle):ab,ti,kw
8.	2 OR 4 OR 5 OR 6 OR 7
9.	8 AND 1
10.	1 AND 3
	9 AND 10
