# Supplementary material for: Postoperative outcomes of preoperative exercise training in patients with operable non-small cell lung cancer: a systematic review and meta-analysis
Source: Front Oncol. 2025 Sep 12;15:1563478. doi: 10.3389/fonc.2025.1563478 (PMC12463627; doi:10.3389/fonc.2025.1563478)
Supplement: Supplementary file 2 [file DataSheet2.pdf]

Supplement Figure 1. Forest plot of the meta-analysis for FEV1

	Preoperative exercise			No preoperative exercise				Mean Difference	
Study or Subgroup	Mean	SD	Total	Mean	SD	Total	Weight	IV, Random, 95% CI	Year
Pehlivan 2011	1.2	0.57	30	0	0.72	30	14.4%	1.20 [0.87, 1.53]	2011
Tereza 2014	0.08	0.53	12	0.24	0.36	12	14.0%	-0.16 [-0.52, 0.20]	2014
Che 2017	0.1	0.5	30	0	0.6	30	15.0%	0.10 [-0.18, 0.38]	2017
Huang 2017 a	0.1	0.6	30	0.1	0.62	30	14.7%	0.00 [-0.31, 0.31]	2017
Huang 2017 b	0	0.8	30	0.1	0.62	30	14.0%	-0.10 [-0.46, 0.26]	2017
Liu 2020	0.13	0.57	37	-0.01	0.53	36	15.3%	0.14 [-0.11, 0.39]	2020
Laurent 2020	0	0.39	14	-0.05	0.75	12	12.6%	0.05 [-0.42, 0.52]	2020
**Total (95% CI)**			**183**			**180**	**100.0%**	**0.18 [-0.16, 0.52]**	

Heterogeneity: Tau² = 0.18; Chi² = 44.68, df = 6 (P < 0.00001); I² = 87%
Test for overall effect: Z = 1.04 (P = 0.30)

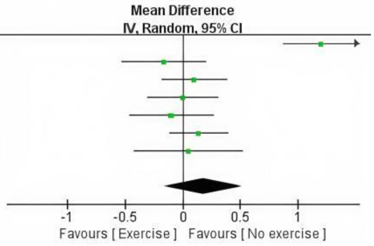

Supplement Figure 2. Forest plot of the meta-analysis for FVC

	Preoperative exercise			No preoperative exercise				Mean Difference	
Study or Subgroup	Mean	SD	Total	Mean	SD	Total	Weight	IV, Fixed, 95% CI	Year
Tereza 2014	0.24	0.85	12	0.1	0.67	12	7.5%	0.14 [-0.47, 0.75]	2014
Che 2017	0.1	0.66	30	0.1	0.7	30	23.7%	0.00 [-0.34, 0.34]	2017
Huang 2017 a	0	0.7	30	-0.1	0.66	30	23.7%	0.10 [-0.24, 0.44]	2017
Huang 2017 b	0	0.85	30	-0.1	0.66	30	18.9%	0.10 [-0.29, 0.49]	2017
Liu 2020	0.16	0.77	37	-0.01	0.65	36	26.3%	0.17 [-0.16, 0.50]	2020
**Total (95% CI)**			**139**			**138**	**100.0%**	**0.10 [-0.07, 0.27]**	

Heterogeneity: Chi² = 0.52, df = 4 (P = 0.97); I² = 0%
Test for overall effect: Z = 1.14 (P = 0.25)

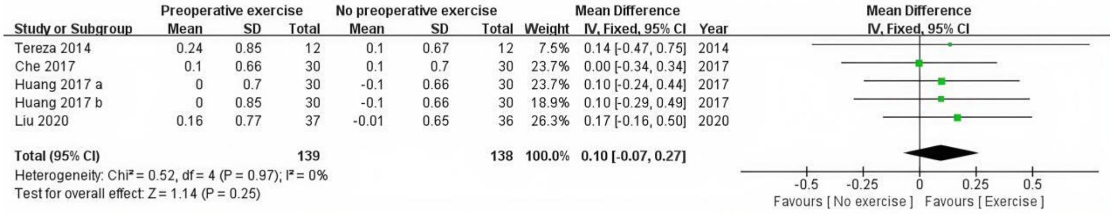

Supplement Figure 3. Forest plot of the meta-analysis for FEV1% of predicted norm values.

	Preoperative exercise			No preoperative exercise				Mean Difference	
Study or Subgroup	Mean	SD	Total	Mean	SD	Total	Weight	IV, Fixed, 95% CI	Year
Pehlivan 2011	15.84	2.1	30	8.92	3.5	30	92.2%	6.92 [5.46, 8.38]	2011
Stefanelli 2013	2.4	19.15	20	-0.1	16.95	20	1.6%	2.50 [-8.71, 13.71]	2013
Tereza 2014	6.7	19.59	12	9.7	12.49	12	1.1%	-3.00 [-16.15, 10.15]	2014
Laurent 2020	-1	13.1	14	-2	26.7	12	0.7%	1.00 [-15.59, 17.59]	2020
Liu 2020	5.4	14.35	37	-1.6	14.67	36	4.4%	7.00 [0.34, 13.66]	2020
**Total (95% CI)**			**113**			**110**	**100.0%**	**6.70 [5.30, 8.10]**	

Heterogeneity: Chi² = 3.18, df = 4 (P = 0.53); I² = 0%
Test for overall effect: Z = 9.36 (P < 0.00001)

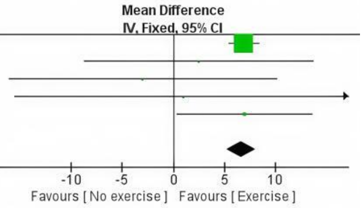

Supplement Figure 4. Forest plot of the meta-analysis for FVC% of predicted norm values.

	Preoperative exercise			No preoperative exercise				Mean Difference	
Study or Subgroup	Mean	SD	Total	Mean	SD	Total	Weight	IV, Fixed, 95% CI	Year
Pehlivan 2011	19.26	2.33	30	16.35	2.4	30	94.7%	2.91 [1.71, 4.11]	2011
Tereza 2014	13.5	16	12	8.5	3.18	12	1.6%	5.00 [-4.23, 14.23]	2014
Liu 2020	5.2	12.44	37	-1.1	13.85	36	3.7%	6.30 [0.26, 12.34]	2020
**Total (95% CI)**			**79**			**78**	**100.0%**	**3.07 [1.90, 4.23]**	

Heterogeneity: Chi² = 1.33, df = 2 (P = 0.51); I² = 0%
Test for overall effect: Z = 5.16 (P < 0.00001)

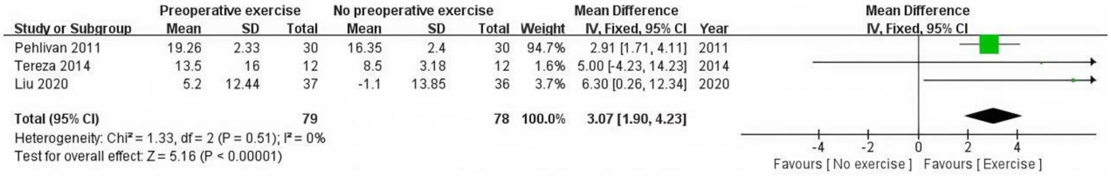

Supplement Figure 5. Forest plot of the meta-analysis for PEF

	Preoperative exercise			No preoperative exercise				Mean Difference	
Study or Subgroup	Mean	SD	Total	Mean	SD	Total	Weight	IV, Fixed, 95% CI	Year
Huang 2017 a	30	114.52	30	-0.3	106	30	18.9%	30.30 [-25.54, 86.14]	2017
Huang 2017 b	75.7	90.91	30	-0.3	106	30	23.5%	76.00 [26.03, 125.97]	2017
Su 2017	25.2	125.06	51	7	90.05	50	32.6%	18.20 [-24.24, 60.64]	2017
Liu 2020	61.8	102.85	37	33.2	108.54	36	25.0%	28.60 [-19.93, 77.13]	2020
**Total (95% CI)**			**148**			**146**	**100.0%**	**36.69 [12.44, 60.93]**	

Heterogeneity: Chi² = 3.26, df = 3 (P = 0.35); I² = 8%
Test for overall effect: Z = 2.97 (P = 0.003)

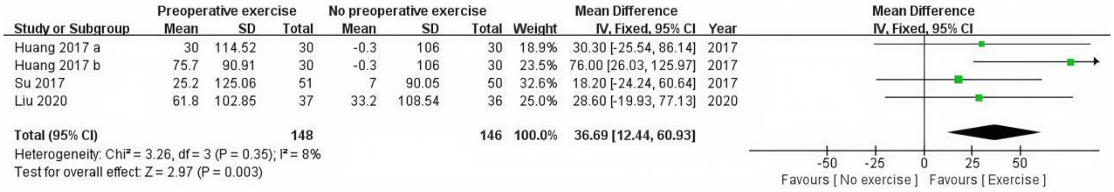

Supplement Figure 6. Forest plot of the meta-analysis for DLCO

Study or Subgroup	Preoperative exercise Mean	SD	Total	No preoperative exercise Mean	SD	Total	Weight	Mean Difference IV, Fixed, 95% CI	Year
Pehlivan 2011	1.93	6.42	30	-1.95	5.34	30	12.5%	3.88 [0.89, 6.87]	2011
Stefanelli 2013	0.3	4.7	20	0.3	2.51	20	20.5%	0.00 [-2.34, 2.34]	2013
Che 2017	0.6	4.53	30	0.3	3.8	30	24.9%	0.30 [-1.82, 2.42]	2017
Huang 2017 a	0.3	4.49	30	-0.5	4.43	30	21.9%	0.80 [-1.46, 3.06]	2017
Huang 2017 b	0.2	4.85	30	-0.5	4.43	30	20.2%	0.70 [-1.65, 3.05]	2017
**Total (95% CI)**			**140**			**140**	**100.0%**	**0.88 [-0.18, 1.93]**	

Heterogeneity: Chi² = 4.73, df = 4 (P = 0.32); I² = 15%
Test for overall effect: Z = 1.63 (P = 0.10)

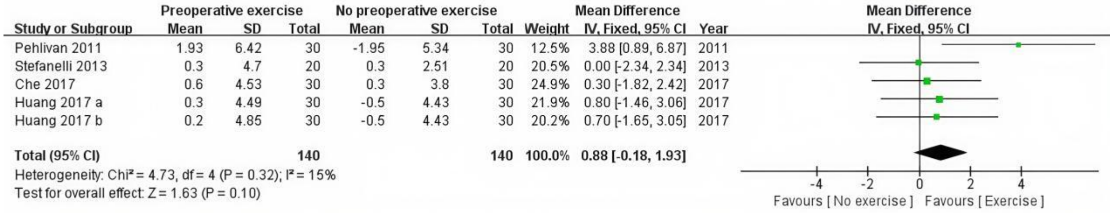

Supplement Figure 7. Forest plot of the meta-analysis for 6MWD

Study or Subgroup	Preoperative exercise Mean	SD	Total	No preoperative exercise Mean	SD	Total	Weight	Mean Difference IV, Random, 95% CI	Year
Tereza 2014	50	85.76	12	-4	107.5	12	10.2%	54.00 [-23.81, 131.81]	2014
Huang 2017 a	36.6	100.49	30	4	84.21	30	12.7%	32.60 [-14.32, 79.52]	2017
Huang 2017 b	6.2	87.78	30	4	84.21	30	12.9%	2.20 [-41.33, 45.73]	2017
Su 2017	23.2	103.87	51	104.2	82.26	50	13.4%	-81.00 [-117.50, -44.50]	2017
Che 2017	28.6	98.52	30	9.36	87.32	30	12.6%	19.24 [-27.87, 66.35]	2017
Bhatia 2019	96	143	74	-32	156.39	77	12.6%	128.00 [80.23, 175.77]	2019
Lai 2019	22.6	116.77	34	2.7	99.71	34	12.3%	19.90 [-31.71, 71.51]	2019
Liu 2020	45.1	98.43	37	3.8	58.29	36	13.4%	41.30 [4.31, 78.29]	2020
**Total (95% CI)**			**298**			**299**	**100.0%**	**25.55 [-18.91, 70.01]**	

Heterogeneity: Tau² = 3493.56; Chi² = 51.85, df = 7 (P < 0.00001); I² = 86%
Test for overall effect: Z = 1.13 (P = 0.26)

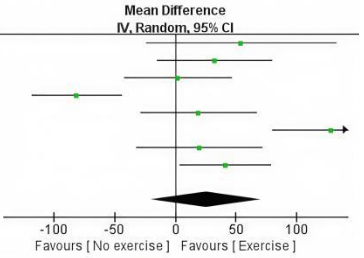

Supplement Figure 8. Forest plot of the meta-analysis for VO2peak

Study or Subgroup	Preoperative exercise Mean	SD	Total	No reoperative exercise Mean	SD	Total	Weight	Mean Difference IV, Fixed, 95% CI	Year
Stefanelli 2013	17.8	2.1	20	14.5	1.2	20	69.5%	3.30 [2.24, 4.36]	2013
Bhatia 2019	22.8	5.7	74	18.9	5.7	77	23.6%	3.90 [2.08, 5.72]	2019
Laurent 2020	18.2	4.32	14	17.1	4.4	12	6.9%	1.10 [-2.26, 4.46]	2020
**Total (95% CI)**			**108**			**109**	**100.0%**	**3.29 [2.41, 4.17]**	

Heterogeneity: Chi² = 2.06, df = 2 (P = 0.36); I² = 3%
Test for overall effect: Z = 7.30 (P < 0.00001)

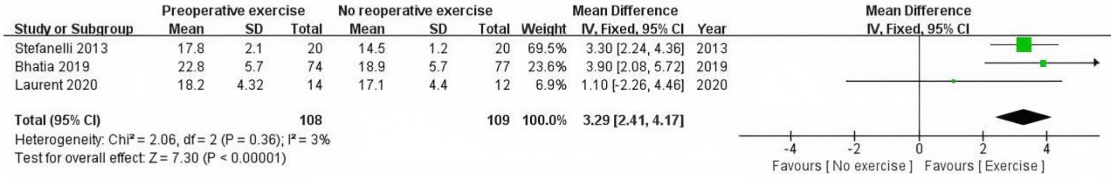

Supplement Figure 9. Forest plot of the meta-analysis for Postoperative severe complications (Clavien-Dindo score ≥2).

Study or Subgroup	Preoperative exercise Events	Total	No preoperative exercise Events	Total	Weight	Odds Ratio M-H, Fixed, 95% CI	Year
Su 2017	5	51	14	50	21.7%	0.28 [0.09, 0.85]	2017
Huang 2017 a	5	30	12	30	17.0%	0.30 [0.09, 1.00]	2017
Huang 2017 b	8	30	12	30	15.0%	0.55 [0.18, 1.62]	2017
Che 2017	6	30	15	30	20.5%	0.25 [0.08, 0.79]	2017
Lai 2019	4	34	12	34	18.0%	0.24 [0.07, 0.86]	2019
Liu 2020	4	37	5	36	7.7%	0.75 [0.18, 3.06]	2020
**Total (95% CI)**		**212**		**210**	**100.0%**	**0.35 [0.21, 0.56]**	
Total events	32		70				

Heterogeneity: Chi² = 2.64, df = 5 (P = 0.76); I² = 0%
Test for overall effect: Z = 4.32 (P < 0.0001)

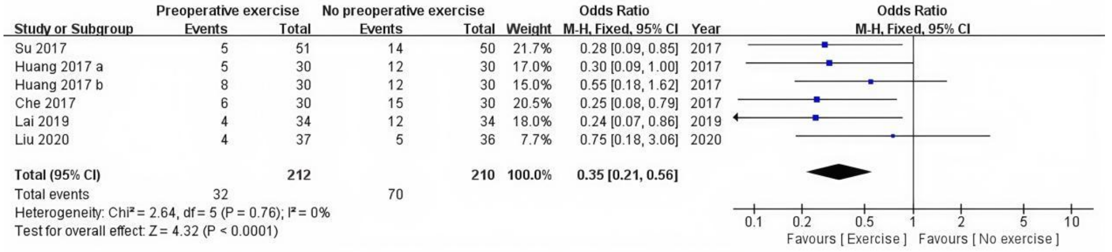

Supplement Figure 10. Forest plot of the meta-analysis for Postoperative 30-day mortality.

Study or Subgroup	Preoperative exercise Events	Total	No preoperative exercise Events	Total	Weight	Odds Ratio M-H, Fixed, 95% CI	Year
Pehlivan 2011	0	30	0	30		Not estimable	2011
Fang 2013	0	22	2	22	33.5%	0.18 [0.01, 4.02]	2013
Che 2017	0	30	1	30	20.2%	0.32 [0.01, 8.24]	2017
Huang 2017 a	0	30	1	30	20.2%	0.32 [0.01, 8.24]	2017
Bhatia 2019	2	74	2	77	26.1%	1.04 [0.14, 7.59]	2019
Lai 2019	0	34	0	34		Not estimable	2019
Liu 2020	0	37	0	36		Not estimable	2020
Zhou 2024	0	51	0	50		Not estimable	2024
**Total (95% CI)**		**308**		**309**	**100.0%**	**0.46 [0.13, 1.67]**	
Total events	2		6				

Heterogeneity: Chi² = 1.08, df = 3 (P = 0.78); I² = 0%
Test for overall effect: Z = 1.18 (P = 0.24)

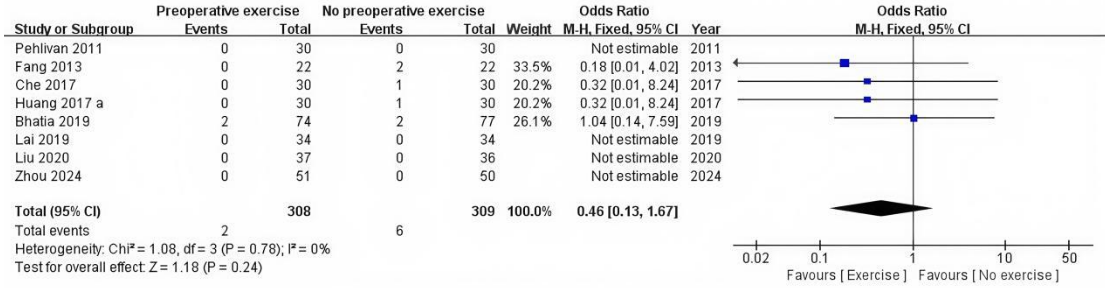

Supplement Figure 11. Forest plot of the meta-analysis for Postoperative chest tube drainage time.

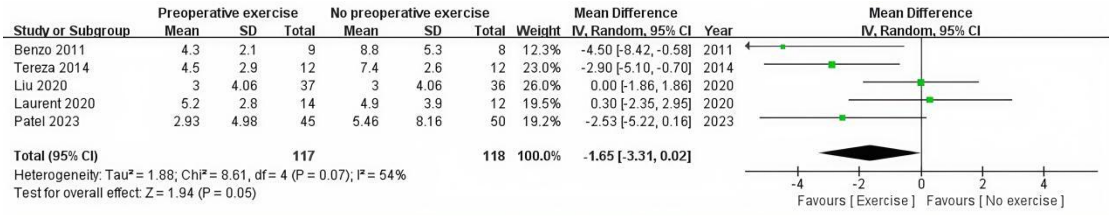

Study or Subgroup	Preoperative exercise Mean	SD	Total	No preoperative exercise Mean	SD	Total	Weight	Mean Difference IV, Random, 95% CI	Year
Benzo 2011	4.3	2.1	9	8.8	5.3	8	12.3%	-4.50 [-8.42, -0.58]	2011
Tereza 2014	4.5	2.9	12	7.4	2.6	12	23.0%	-2.90 [-5.10, -0.70]	2014
Liu 2020	3	4.06	37	3	4.06	36	26.0%	0.00 [-1.86, 1.86]	2020
Laurent 2020	5.2	2.8	14	4.9	3.9	12	19.5%	0.30 [-2.35, 2.95]	2020
Patel 2023	2.93	4.98	45	5.46	8.16	50	19.2%	-2.53 [-5.22, 0.16]	2023
**Total (95% CI)**			**117**			**118**	**100.0%**	**-1.65 [-3.31, 0.02]**	

Heterogeneity: Tau² = 1.88; Chi² = 8.61, df = 4 (P = 0.07); I² = 54%
Test for overall effect: Z = 1.94 (P = 0.05)

Supplement Figure 12. Forest plot of the meta-analysis for Postintervention (preoperative) dyspnoea.

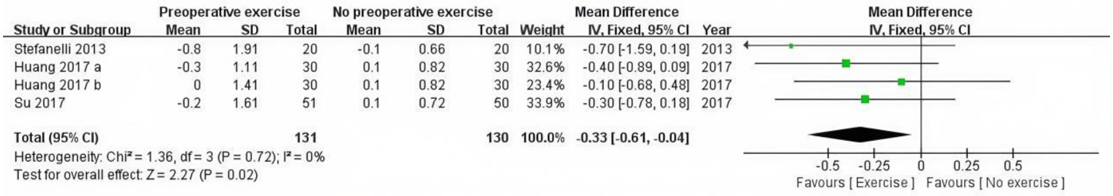

Study or Subgroup	Preoperative exercise Mean	SD	Total	No preoperative exercise Mean	SD	Total	Weight	Mean Difference IV, Fixed, 95% CI	Year
Stefanelli 2013	-0.8	1.91	20	-0.1	0.66	20	10.1%	-0.70 [-1.59, 0.19]	2013
Huang 2017 a	-0.3	1.11	30	0.1	0.82	30	32.6%	-0.40 [-0.89, 0.09]	2017
Huang 2017 b	0	1.41	30	0.1	0.82	30	23.4%	-0.10 [-0.68, 0.48]	2017
Su 2017	-0.2	1.61	51	0.1	0.72	50	33.9%	-0.30 [-0.78, 0.18]	2017
**Total (95% CI)**			**131**			**130**	**100.0%**	**-0.33 [-0.61, -0.04]**	

Heterogeneity: Chi² = 1.36, df = 3 (P = 0.72); I² = 0%
Test for overall effect: Z = 2.27 (P = 0.02)

Supplement Figure 13. Forest plot of the meta-analysis for Postintervention (preoperative) Health-related quality of life (HRQoL).

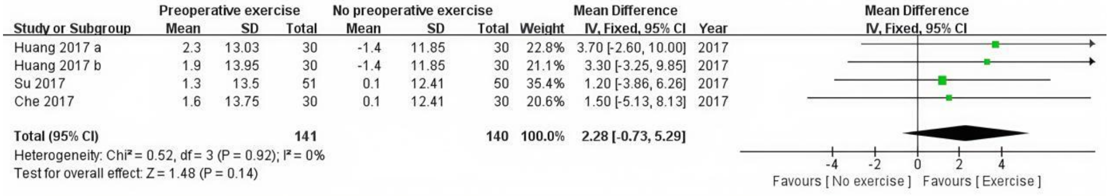

Study or Subgroup	Preoperative exercise Mean	SD	Total	No preoperative exercise Mean	SD	Total	Weight	Mean Difference IV, Fixed, 95% CI	Year
Huang 2017 a	2.3	13.03	30	-1.4	11.85	30	22.8%	3.70 [-2.60, 10.00]	2017
Huang 2017 b	1.9	13.95	30	-1.4	11.85	30	21.1%	3.30 [-3.25, 9.85]	2017
Su 2017	1.3	13.5	51	0.1	12.41	50	35.4%	1.20 [-3.86, 6.26]	2017
Che 2017	1.6	13.75	30	0.1	12.41	30	20.6%	1.50 [-5.13, 8.13]	2017
**Total (95% CI)**			**141**			**140**	**100.0%**	**2.28 [-0.73, 5.29]**	

Heterogeneity: Chi² = 0.52, df = 3 (P = 0.92); I² = 0%
Test for overall effect: Z = 1.48 (P = 0.14)
